# Transcranial direct current stimulation (tDCS) for improving capacity in activities and arm function after stroke: a network meta-analysis of randomised controlled trials

**DOI:** 10.1186/s12984-017-0301-7

**Published:** 2017-09-13

**Authors:** Bernhard Elsner, Gert Kwakkel, Joachim Kugler, Jan Mehrholz

**Affiliations:** 1 Department of Public Health, Dresden Medical School, Technical University Dresden, Fetscherstr. 74, 01307 Dresden, Germany; 2 Physiotherapy, SRH University of Applied Health Sciences Gera, Gera, Germany; 3 Department of Rehabilitation Medicine, VU University Medical Center, MOVE Research Institute Amsterdam, Amsterdam, The Netherlands; 4 Neurorehabilitation, Amsterdam Rehabilitation Research Center Reade, Amsterdam, The Netherlands; 5 Neuroscience Campus Amsterdam, VU University Amsterdam, Amsterdam, The Netherlands; 6 Department of Physical Therapy and Human Movement Sciences, Northwestern University, Evanston, IL USA; 7 Wissenschaftliches Institut, Private Europäische Medizinische Akademie der Klinik Bavaria in Kreischa GmbH, Kreischa, Germany

**Keywords:** Stroke, Recovery of function, Transcranial direct current stimulation, Review, Meta-analysis

## Abstract

**Background:**

Transcranial Direct Current Stimulation (tDCS) is an emerging approach for improving capacity in activities of daily living (ADL) and upper limb function after stroke. However, it remains unclear what type of tDCS stimulation is most effective. Our aim was to give an overview of the evidence network regarding the efficacy and safety of tDCS and to estimate the effectiveness of the different stimulation types.

**Methods:**

We performed a systematic review of randomised trials using network meta-analysis (NMA), searching the following databases until 5 July 2016: Cochrane Central Register of Controlled Trials (CENTRAL), MEDLINE, EMBASE, CINAHL, AMED, Web of Science, and four other databases. We included studies with adult people with stroke. We compared any kind of active tDCS (anodal, cathodal, or dual, that is applying anodal and cathodal tDCS concurrently) regarding improvement of our primary outcome of ADL capacity, versus control, after stroke. PROSPERO ID: CRD42016042055.

**Results:**

We included 26 studies with 754 participants. Our NMA showed evidence of an effect of cathodal tDCS in improving our primary outcome, that of ADL capacity (standardized mean difference, SMD = 0.42; 95% CI 0.14 to 0.70). tDCS did not improve our secondary outcome, that of arm function, measured by the Fugl-Meyer upper extremity assessment (FM-UE). There was no difference in safety between tDCS and its control interventions, measured by the number of dropouts and adverse events.

**Conclusion:**

Comparing different forms of tDCS shows that cathodal tDCS is the most promising treatment option to improve ADL capacity in people with stroke.

**Electronic supplementary material:**

The online version of this article (10.1186/s12984-017-0301-7) contains supplementary material, which is available to authorized users.

## Background

An emerging approach for enhancing neural plasticity and hence rehabilitation outcomes after stroke is non-invasive brain stimulation (NIBS). Several stimulation procedures are available, such as repetitive transcranial magnetic stimulation (rTMS) [1], transcranial direct current stimulation (tDCS) [2–4], transcranial alternating current stimulation (tACS) [5], and transcranial pulsed ultrasound (TPU) [6]. In recent years a considerable evidence base for NIBS has emerged, especially for rTMS and tDCS.

tDCS is relatively inexpensive, easy to administer and portable, hence constituting an ideal adjuvant therapy during stroke rehabilitation. It works by applying a weak and constant direct current to the brain and has the ability to either enhance or suppress cortical excitability, with effect lasting up to several hours after the stimulation [7–9]. Hypothetically, this technique makes tDCS a potentially useful tool to modulate neuronal inhibitory and excitatory networks of the affected and the non-affected hemisphere post stroke to enhance, for example, upper limb motor recovery [10, 11]. Three different stimulation types can be distinguished.

- In anodal stimulation, the anodal electrode (+) usually is placed over the lesioned brain area and the reference electrode over the contralateral orbit [12]. This leads to subthreshold depolarization, hence promoting neural excitation [3].
- In cathodal stimulation, the cathode (−) usually is placed over the non-lesioned brain area and the reference electrode over the contralateral orbit [12], leading to subthreshold polarization and hence inhibiting neural activity [3].
- Dual tDCS means the simultaneous application of anodal and cathodal stimulation [13].

However, the literature does not provide clear guidelines, not only regarding the tDCS type, but also regarding the electrode configuration [14], the amount of current applied and the duration of tDCS, or the question if tDCS should be applied as a standalone therapy or in combination with other treatments, like robot-assisted therapy [15].

### Rationale

There is so far conflicting evidence from systematic reviews of randomised controlled trials on the effectiveness of different tDCS approaches after stroke. For example, over the past two decades more than 30 randomised clinical trials have investigated the effects of different tDCS stimulation techniques for stroke, and there are 55 ongoing trials [16]. However, the resulting network of evidence from randomised controlled trials (RCTs) investigating different types of tDCS (i.e., anodal, cathodal or dual) as well as their comparators like sham tDCS, physical rehabilitation or pharmacological agents has not yet been analyzed in a systematic review so far.

A network meta-analysis (NMA), also known as multiple treatment comparison meta-analysis or mixed treatment comparison analysis, allows for a quantitative synthesis of the evidence network. This is made possible by combining direct evidence from head-to-head comparisons of three or more interventions within randomised trials with indirect evidence across randomised trials on the basis of a common comparator [17–20]. Network meta-analysis has many advantages over traditional pairwise meta-analysis, such as visualizing and facilitating the interpretation of the wider picture of the evidence and improving understanding of the relative merits of these different types of neuromodulation when compared to sham tDCS and/or another comparator such as exercise therapy and/or pharmacological agents [21, 22]. By borrowing strength from indirect evidence to gain certainty about all treatment comparisons, network meta-analysis allows comparative effects that have not been investigated directly in randomised clinical trials to be estimated and ranked [22, 23].

### Objective

The aim of our systematic review with NMA was to give an overview of the evidence network of randomised controlled trials of tDCS (anodal, cathodal, or dual) for improving capacity in activities of daily living (ADL) and upper limb function after stroke, as well as its safety, and to estimate and rank the relative effectiveness of the different stimulation types, while taking into account potentially important treatment effect modifiers.

## Methods

### Protocol and registration

We published a study protocol, which has been registered in the PROSPERO database under the ID CRD42016042055. Our protocol adheres to the PRISMA extension statement for NMA [24].

### Role of the funding source

There was no funding source for this study.

### Eligibility criteria

We included studies with adults who had experienced a stroke. We compared any kind of active tDCS (anodal, cathodal, or dual, that is applying anodal and cathodal tDCS concurrently) for improving our primary outcome of ADL capacity and our secondary outcome of arm function after stroke. Another secondary outcome was safety, measured by the number of dropouts and adverse events. We defined active tDCS as any application of direct current to the skull lasting longer than 1 min. This is approximately the time it takes to fade in and fade out the sham application of tDCS in order to produce perceivable sensations on the skin similar to active tDCS [25]. We included all studies with outcome measures evaluating ADL capacity, and for arm motor function we included all studies using the Fugl-Meyer upper extremity assessment (UE-FM). We included all genuine RCTs and genuine randomised controlled cross-over trials which compared tDCS with any other intervention. We analyzed only the first intervention phase of trials with a cross-over design, and assumed between-group differences to be identical to those in trials with a parallel group design. We combined different stimulation durations, different electrical currents applied and different stimulation locations for the same stimulation type (that is anodal, cathodal, or dual tDCS).

### Information sources

We searched the following databases until 5 July 2016: Cochrane Central Register of Controlled Trials (CENTRAL; the Cochrane Library; 2016, Issue 7), MEDLINE (from 1948), EMBASE (from 1980), CINAHL (from 1982), AMED (from 1985), Web of Science (from 1899), Physiotherapy Evidence Database, Rehabdata, COMPENDEX (from 1969) and INSPEC (from 1969). There were no language restrictions. In order to identify further published and unpublished trials, we searched trial registers and reference lists, hand-searched conference proceedings and contacted authors and equipment manufacturers.

### Search

The search strategy for MEDLINE can be found in Additional file 1. This search strategy was adapted for the other databases.

### Study selection

One review author (BE) excluded obviously irrelevant studies by reading titles and abstracts. We retrieved the full text of the remaining studies, which were then ranked by two review authors (JK, BE) as relevant, possibly relevant, or irrelevant according to our inclusion criteria. Two review authors (JK, JM) decided whether the possibly relevant publications fitted the PICOS strategy (Patient, Intervention, Comparison, Outcome, Study type) of our research question. We excluded all trials ranked as irrelevant and tested all trials ranked as relevant or possibly relevant for inclusion. Disagreements were resolved by discussions with all review authors.

### Data collection process

Two review authors (BE, JM) independently extracted trial and summary outcome data from the trials.

### Data items

We used checklists to independently assess the following items: (1) methods of random sequence generation, (2) methods of allocation concealment, (3) blinding of outcome assessors, participants and personnel, (4) use of an intention-to-treat analysis, (5) adverse effects and dropouts, (6) important differences in prognostic factors, (7) participants (number, age, time from stroke onset to study entry), (8) comparison (details of interventions in treatment and control groups, duration of treatment and details of co-interventions in the groups) and (9) outcomes at the end of the study.

### Geometry of the network

The geometry of the network characterizes the relation and precision of direct comparisons. At the level of type of intervention we analyzed what type of tDCS (anodal, cathodal, or dual) was compared with a particular control intervention. The geometry of the network was assessed by generating network graphs [26]. Each type of intervention represents a node in the network. Randomised comparisons between interventions are shown as links between the nodes. Multi-arm studies are indicated by colored polygons [27].

### Risk of bias within individual studies

We assessed risk of bias of included studies by the Cochrane risk of bias tool at study and at outcome level [28]. The results were incorporated into our sensitivity analysis, where only studies with low risk of bias were included. We presented the results for each outcome in a separate figure.

### Summary measures

Considering studies that used the same outcome measure for an outcome, we calculated Mean Differences (MD) and their corresponding 95% Confidence Intervals (CI). Including studies that did not use the same outcome measure, but did measure the same underlying construct, we calculated Standardized Mean Differences (SMD) and their corresponding 95% CIs. We expected the number of dropouts and adverse events to be rare and therefore calculated the Risk Difference (RD) and corresponding 95% CIs as the summary measure. For all comparisons we generated contrast-based forest plots. We performed a relative ranking of the competing interventions according to their P-scores [27]. The P-score of an intervention, which may range from 0 to 1 and, can be interpreted as the mean certainty of its superiority and thus is comparable to its surface under the cumulative ranking curve (SUCRA) [26, 29]. All statistical analyses have been performed with the statistical software R version 3.2.2 [30], package “netmeta” version 0.8–0 [27].

### Planned method of analysis

This network meta-analysis was based on a frequentist weighted least squares approach [31, 32], which is roughly equivalent to maximum likelihood estimation [29]. This approach follows the graph-theoretical methodology and allows incorporation of multi-arm trials [32]. The analysis is based on two assumptions: (a) independence of studies and (b) consistency of the underlying effects (transitivity assumption) [29]. We considered the treatment effects to differ between the proof-of-concept trials, and therefore applied a random-effects model to estimate summary treatment effects, based on treatment contrasts.

### Assessment of inconsistency

As proposed by Higgins et al. for network meta-analyses, we used a generalized Cochran’s Q statistic for multivariate meta-analysis to test the homogeneity and inconsistency assumptions [33, 34]. This Q statistic contains one within-design Q statistic and one between-design Q statistic, the latter being calculated on the basis of a full design-by-treatment interaction random-effects model, thus embracing the concept of design inconsistency [34]. We assessed inconsistency locally (that is between pairwise comparisons) using the net heat plot [34]. The net heat plot is based on a global chi-squared test for inconsistency based on the comparison of an inconsistency model and a consistency model [34]. It identifies inconsistency between specific direct evidence in the network and provides possible drivers [34].

### Risk of bias across studies

We assessed the risk of bias regarding selective reporting by comparing the published protocol of a study with the corresponding full text obtained by our electronic search.

### Additional analyses

We considered allocation concealment, blinding of outcome assessor and intention-to-treat analysis to be potentially important effect modifiers and incorporated them in our sensitivity analysis. Although we conducted random-effects analyses regardless of the level of heterogeneity, we defined the choice of analysis method (fixed-effect versus random-effects model) a priori as another potentially important effect modifier, and therefore assessed the effect of heterogeneity on estimated treatment comparisons by visually inspecting Bland-Altman plots [29].

We also conducted a post-hoc meta-regression analysis of all sham-controlled studies in order to identify low- and high-inference moderator variables (i.e., codings) that may have biased outcomes of ADL capacity or arm function, as measured by UE-FM [35]. For the high-interference codings, we investigated the impact of the publication year to investigate novelty effects. For low-interference codings based on information of the trial itself, we investigated factors such as time since stroke, electric current [mA], duration of stimulation session [min], number of stimulation sessions, electrode size [cm^2^], current density [mA/cm^2^], electric charge per session [C], and charge density per session [C/cm^2^]. We used the simultaneous entry approach to fit the meta-regression model [36].

## Results

### Study selection

**Fig. 1:**
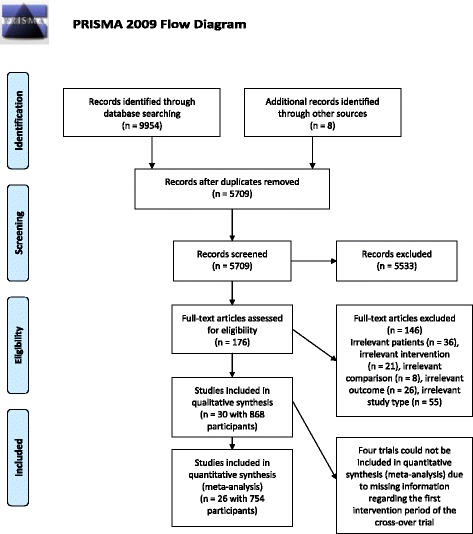
Study flow diagram

We screened 5709 unique records and assessed 176 full-text articles for eligibility. We included 30 trials with 868 participants in a qualitative analysis and 26 studies with 754 participants in a quantitative synthesis (meta-analysis). Figure 1 shows the flow of studies.

### Study characteristics

Twenty-four of the 30 studies (80%) were RCTs, and the remaining six studies (20%) were randomised crossover trials. The sample sizes of the included studies ranged from four [37] to 96 [38]. The mean (SD) sample size was 26 (22) with a median sample size of 20. The mean age in the experimental groups ranged from 43 to 67 years and that in the control groups from 45 to 75 years. The mean time since stroke ranged from 3 days to 8 years. The current applied ranged from 1 mA to 2 mA, while the number of stimulation sessions ranged from five to 30.

A comprehensive summary of the characteristics of the included trials examining tDCS for improving ADL capacity and upper limb function, and its safety, can be found in Additional file 2.

### Presentation of network structure

**Fig. 2:**
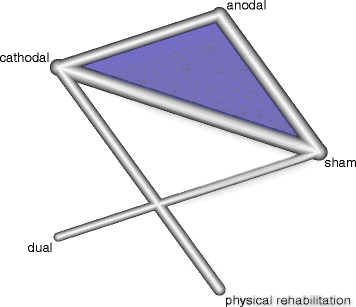
Network graph of tDCS for improving ADL capacity after stroke. The thicker the edge, the lower the standard error of this comparison. Colored polygons indicate multi-arm studies

**Fig. 3:**
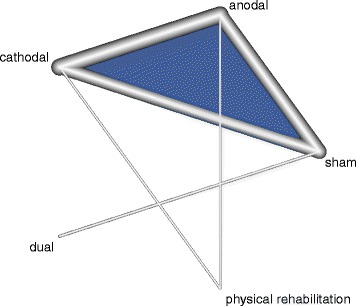
Network graph of tDCS for improving arm function (measured by UE-FM) after stroke. The thicker the edge, the lower the standard error of this comparison. Colored polygons indicate multi-arm studies. UE-FM: Upper Extremity Fugl-Meyer Assessment

**Fig. 4:**
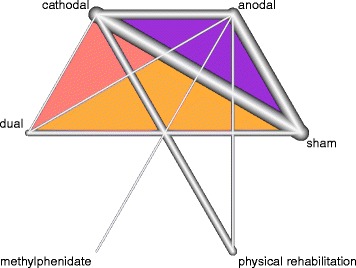
Network graph of the safety of tDCS (measured by number of dropouts and adverse events) after stroke. The thicker the edge, the lower the standard error of this comparison. Colored polygons indicate multi-arm studies

Figure 2 shows a network graph comparing anodal, cathodal, and dual tDCS with their control interventions for improving ADL capacity after stroke. Figure 3 shows a network graph comparing anodal, cathodal, and dual tDCS with their control interventions for improving arm function (measured by the upper extremity UE-FM) after stroke. Figure 4 shows a network graph comparing tDCS with their control interventions regarding safety (measured by the number of dropouts and adverse events).

### Summary of network geometry

A total of 284 patients received active tDCS to improve their ADL capacity (number of studies = 12, number of study arms = 27) [38–49]. The intervention types studied were mostly cathodal tDCS (seven study arms with 167 participants) [38, 43, 44, 46, 49–51], anodal tDCS (six study arms with 88 participants) [38, 39, 43, 46, 48, 52–56], or dual tDCS (three study arms with 29 participants) [40, 41, 47]. A total of 163 participants received sham tDCS as a comparator intervention (number of studies = 10) [37–41, 44–46]. Two study arms with 45 participants used physical rehabilitation comparators like virtual reality and physical therapy [44, 45].

A total of 302 patients received active tDCS to improve their arm function, as measured by UE-FM (number of studies = 16, number of study arms = 35) [38, 39, 43, 44, 46–57]. The intervention types studied were mostly cathodal tDCS (seven study arms with 140 participants) [38, 43, 44, 46, 49–51], anodal tDCS (ten study arms with 140 participants) [38, 39, 43, 46, 48, 52–56], or dual tDCS (two study arms with 22 participants) [47, 57]. A total of 214 participants received sham tDCS as a comparator intervention (number of study arms = 14) [38, 39, 43, 46–51, 53–57]. Two study arms with 30 participants used physical rehabilitation comparators like virtual reality and physical therapy [44, 52].

We identified 26 trials (number of study arms = 57) with 754 participants which investigated tDCS for improving ADL capacity or arm function and extracted data regarding the safety of tDCS (number of dropouts and adverse effects) [37–47, 49–56, 58–62]. The intervention types studied were mostly anodal tDCS (16 study arms with 174 participants) [37–39, 42, 43, 46, 48, 52–56, 58, 60–62], cathodal tDCS (11 study arms with 170 participants) [37, 38, 42–46, 49, 51, 58, 59], and dual tDCS (seven study arms with 65 participants) [40, 41, 47, 57, 58, 61, 63].

Additional file 3 shows the risk of bias within studies of tDCS for improving ADL capacity, arm function, and safety.

Additional file 4 shows a possible approach to presenting data from studies examining the effects of tDCS on ADL capacity and arm function, as well as the safety of the interventions (anodal, cathodal, dual or sham tDCS, physical rehabilitation interventions and methylphenidate).

### Synthesis of results

**Fig. 5:**
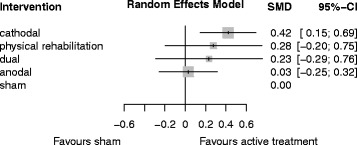
Forest plot of tDCS for improving ADL capacity after stroke (12 studies with 284 participants). Treatments are listed in order of relative ranking. SMD = standardized mean difference, CI = confidence interval. Sham is the reference category

Table 1 provides a comparison of effect estimates obtained from the network meta-analysis with effect estimates obtained from direct comparisons by pairwise meta-analysis of tDCS for improving ADL capacity. Table 2 shows the ranking of the treatments by P-score, and Fig. 5 shows the forest plot of tDCS for improving ADL capacity after stroke.

**Table 1:** League table for comparing network estimates with direct estimates of tDCS for improving ADL capacity

Cathodal	0.15 (−0.2; 0.5)	–	0.43 (0.0; 0.8)	0.1 (−0.3; 0.4)
0.14 (−0.2; 0.5)	Physical rehabilitation	–	–	–
0.19 (−0.4; 0.8)	0.04 (−0.7; 0.8)	Dual	0.23 (−0.3; 0.8)	–
0.39 (0.1; 0.7)	0.25 (−0.3; 0.8)	0.25 (−0.4; 0.9)	Anodal	0.13 (−0.2; 0.5)
0.42 (0.2; 0.7)	0.28 (−0.2; 0.8)	0.23 (−0.8; 0.3)	0.03 (−0.3; 0.3)	Sham

League table for comparing network estimates (lower triangle) with direct estimates of pairwise meta-analysis (upper triangle) of tDCS for improving ADL capacity (SMD and corresponding 95% CI). Treatments are listed in order of relative ranking. Comparisons between treatments should be read from left to right. Their SMD and corresponding 95% CI can be obtained from the cell shared by the column defining treatment and the row defining treatment. Positive SMDs favor the column defining treatment for the network estimates (lower triangle) and the row defining treatment for the direct estimates (upper triangle). Physical rehabilitation means control interventions like physiotherapy, occupational therapy, or virtual reality training

**Table 2:** treatment rankings by P-score of tDCS for improving ADL capacity

Treatment	P-Score
Cathodal	0.87
Physical rehabilitation	0.62
Dual	0.57
Anodal	0.25
Sham	0.18

Treatments are listed in order of relative ranking. The P-Score, ranging from 0 to 1, describes the mean degree of certainty about a particular treatment being better than another treatment

Table 3 provides a comparison of effect estimates obtained from the network meta-analysis with effect estimates obtained from direct comparisons by pairwise meta-analysis for improving arm function after stroke.

**Table 3:** League table for comparing network estimates with direct estimates of tDCS for improving arm function

Cathodal	–	−0.63 (−5.4; 4.2)	4.35 (0.6; 8.1)	3.76 (−7.9; 15.4)
0.25 (−11.7;12.2)	Dual	–	2.47 (−6.0; 11.0)	–
0.93 (−4.0; 5.9)	0.67 (−11.1; 12.5)	Anodal	1.45 (−3.5; 6.4)	19.00 (9.4; 28.6)
2.67 (−2.7; 9.0)	2.4 (−8.6; 13.5)	0.67 (−12.5; 11.1)	Sham	–
13.48 (−1.8; 7.2)	12.44 (−3.0; 27.8)	11.8 (1.38; 22.1)	10.02 (0.72; 20.8)	Physical rehabilitation

League table to compare network estimates (lower triangle) with direct estimates of pairwise meta-analysis (upper triangle) of tDCS for improving arm function (MD and corresponding 95% CI). Treatments are listed in order of relative ranking. Comparisons between treatments should be read from left to right. Their MD (unit: UE-FM scores) and corresponding 95% CI can be obtained from the cell shared by the column defining treatment and the row defining treatment. Positive MDs favor the column defining treatment for the network estimates (lower triangle) and the row defining treatment for the direct estimates (upper triangle). Physical rehabilitation means control interventions like physiotherapy, occupational therapy or virtual reality training. UE-FM: Upper Extremity Fugl-Meyer assessment

**Fig. 6:**
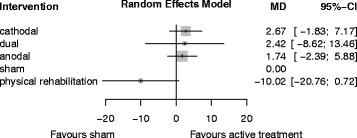
Forest plot of tDCS for improving arm function after stroke (16 studies with 302 participants). Treatments are listed in order of relative ranking. MD = mean difference [UE-FM points], CI = confidence interval. Sham is the reference category

Table 4 shows the ranking of treatments by P-score, and Fig. 6 shows the forest plot of tDCS for improving arm function after stroke.

**Table 4:** Treatment rankings by P-score of tDCS for improving arm function

Treatment	P-Score
Cathodal	0.76
Dual	0.66
Anodal	0.65
Sham	0.41
Physical rehabilitation	0.03

Treatments are listed in order of relative ranking. The P-Score, ranging from 0 to 1, describes the mean degree of certainty about a particular treatment being better than another treatment

**Fig. 7:**
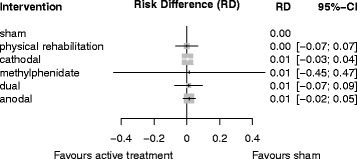
Forest plot of the safety of tDCS for improving ADL capacity or arm function after stroke (26 studies with 754 participants). Treatments are listed in order of relative ranking. RD = Risk Difference, CI = confidence interval. Sham is the reference category

Table 5 provides a comparison of effect estimates obtained from the network meta-analysis with effect estimates obtained from direct comparisons by pairwise meta-analysis. Table 6 shows the ranking of treatments by P-score, and Fig. 7 shows the forest plot of the safety of tDCS for improving ADL capacity and arm function after stroke.

**Table 5:** League table for comparing network estimates with direct estimates of tDCS for safety of tDCS

Sham	–	0.00 (−0.9; 0.1)	–	0.01 (−0.1; 0.1)	0.01 (−0.0; 0.1)
0.00 (−0.1; 0.1)	Physical rehabilitation	0.01 (−0.1; 0.0)	–	–	0.00 (−0.2; 0.2)
0.00 (−0.0; 0.0)	0.00 (−0.1; 0.1)	Cathodal	–	0.00 (−0.7; 0.7)	0.00 (−0.1; 0.1)
0.01 (−0.5; 0.5)	0.01 (−0.5; 0.5)	0.01 (−0.5; 0.5)	Methylphenidate		0.00 (−0.5; 0.5)
0.01 (−0.1; 0.1)	0.01 (−0.1; 0.1)	0.01 (−0.1; 0.1)	0.00 (−0.5; 0.5)	Dual	0.00 (−0.2; 0.2)
0.01 (−0.1;0.0)	0.01 (−0.1; 0.1)	0.01 (−0.0; 0.0)	0.00 (−0.5; 0.5)	0.00 (−0.1; 0.1)	Anodal

League table for comparing network estimates (lower triangle) with direct estimates of pairwise meta-analysis (upper triangle) of the safety of tDCS (measured by drop-outs and adverse events during intervention phase) (RD and corresponding 95% CI). Treatments are listed in order of relative ranking. Comparisons between treatments should be read from left to right. Their RD and corresponding 95% CI can be obtained from the cell shared by the column defining treatment and the row defining treatment. Positive RDs favor the column defining treatment for the network estimates (lower triangle) and the row defining treatment for the direct estimates (upper triangle). Physical rehabilitation means control interventions like physiotherapy, occupational therapy, or virtual reality training

**Table 6:** Treatment rankings by P-score of the safety of tDCS (measured by drop-outs and adverse events during the intervention phase)

Treatment	P-Score
Sham	0.60
Physical rehabilitation	0.57
Cathodal	0.50
Methylphenidate	0.49
Dual	0.46
Anodal	0.38

Treatments are listed in order of relative ranking. The P-Score, ranging from 0 to 1, describes the mean degree of certainty about a particular treatment being better than another treatment

### Exploration for inconsistency

Significant inconsistency, which means disagreement between direct and indirect comparisons, was not observed. Visual inspection of the net heat plots of the three comparisons did not yield excess inconsistency, and formal testing did not detect statistically significant design inconsistency (Q = 1.29; df = 2; *p* = 0.52 for ADL capacity; Q = 7.3; df = 3; *p* = 0.06 for arm function, and Q = 0.88; df = 2; *p* = 0.66 for safety). The accompanying net heat plots can be found in Additional file 5.

### Risk of bias across studies

We assessed the risk of bias qualitatively for each outcome by visual inspection of its risk-of-bias graph. We assessed the risk of bias regarding tDCS for improving ADL capacity and arm function and its safety as low to unclear. The risk-of-bias graphs can be found in Additional file 3.

### Results of additional analyses

Our prespecified sensitivity analysis on the effects of methodological quality regarding our primary outcome measure (ADL capacity) included four studies with 247 participants with proper allocation concealment, blinding of outcome assessor and intention-to-treat analysis. Cathodal tDCS remained the most effective stimulation type (Standardized Mean Difference, SMD = 0.4; 95% CI: 0.1 to 0.7). The full results of our subgroup analysis can be found in Additional file 6.

As prespecified in our protocol, we used random-effects models for each comparison, and additionally compared them with fixed-effects models in order to assess the effect of heterogeneity on estimated treatment outcomes. Visual inspection of Bland-Altman plots did not yield any systematic differences between the results of fixed- and random-effects models for any of the outcomes. The results of our sensitivity analyses can be found in Additional file 6.

We could not identify any statistically significant effect moderators of tDCS for improving ADL capacity or arm function. The detailed results of our meta-regression analyses can be found in Additional file 7.

## Discussion

This systematic review with a network meta-analysis included 12 randomized controlled trials with 284 participants examining the effect of tDCS on our primary outcome, that of ADL capacity. We found evidence of a significant moderate effect in favor of cathodal tDCS, whereas no significant effects were found for the other active tDCS (i.e., dual tDCS, anodal tDCS, and sham tDCS) or control interventions. Sixteen studies with 302 participants examined our secondary outcome, that of upper limb motor function as measured with Upper Extremity Fugl-Meyer Motor scores (UE-FM). We found no evidence of an effect of cathodal tDCS, dual tDCS, anodal tDCS, sham tDCS, or physical rehabilitation interventions. Finally, our analysis of 26 trials with 754 participants found no statistically significant differences between sham tDCS, physical rehabilitation interventions, cathodal tDCS, methylphenidate, dual tDCS, and anodal tDCS, regarding our other secondary outcome, that of the safety of tDCS or its control interventions as revealed by the number of dropouts and adverse events.

The results of this network meta-analysis in terms of our primary outcome, ADL capacity, are in line with a recent Cochrane review examining the effects of tDCS in improving activities, arm and lower extremity function, muscle strength, and cognition [16]. Due to the methodological limitations inherent in traditional pairwise meta-analyses the authors of that review could only draw pairwise comparisons, not taking into account the existing evidence network. Furthermore, in order to avoid multiple testing, the authors had to combine treatment groups with different types of tDCS into a single tDCS group, thus maybe masking possible differences between different tDCS types. In a pre-specified formal subgroup analysis for their primary outcome of activities, the authors tried to estimate the treatment effects of the different tDCS types (anodal, cathodal, and dual tDCS). The analysis suggested a favorable effect of cathodal tDCS for improving ADL after stroke (SMD 0.33, 95% CI 0.10 to 0.57; six studies with 301 participants), whereas there was no effect for anodal (SMD -0.04, 95% CI -0.35 to 0.27; five studies with 164 participants) or dual tDCS (SMD 0.30, 95% CI -0.39 to 0.99; two studies with 33 participants), which is in accordance with our findings.

The relative superiority of cathodal tDCS might be due to a downregulation of the overactive non-affected brain hemisphere as a result of insufficient interhemispheric inhibition and with that, restoring the balance of excitatory and inhibitory interactions between both hemispheres [64–67]. From this point of view, cathodal tDCS should rather be regarded as supporting the downregulation of overactivity of the non-lesioned hemisphere. This might contrast with the model of ‘vicariation of function’ [68, 69] which suggests that unaffected brain areas ‘take over’ functions of the affected hemisphere [64, 69]. Recently, doubts have been raised about whether this model may be oversimplified or even incorrect and new models have been proposed, such as the bimodal balance-recovery model, which links interhemispheric balancing to the brain’s remaining structural reserve [64].

The optimal stimulation paradigm, in terms of the selection of participants likely to benefit from tDCS, the electrode size and location, the amount of direct current applied and the duration of administration remains to be established [14, 64, 70]. Besides the above-mentioned neurophysiological explanation for the finding of superiority of cathodal tDCS, there might also be methodological reasons. For example, the majority of participants in randomised studies of tDCS for improving ADL capacity were treated with cathodal tDCS (167 out of 284 participants, 59%). Hence, this intervention might have the greatest statistical power in showing evidence, whereas dual tDCS was the least powered intervention.

Regarding our secondary outcome (i.e., function of the upper paretic limb), our results are in line with two systematic reviews with pairwise meta-analysis. Tedesco Triccas and colleagues (2015) included genuine RCTs with multiple sessions of tDCS for improving the function of the upper paretic limb after stroke [71]. They included nine studies with 371 participants. Their analysis did not reveal any statistically significant effect of active tDCS at the end of the intervention period (SMD 0.11, 95% CI -0.17 to 0.38). The other systematic review of the effects of anodal tDCS on upper extremity function and cortical excitability in people with stroke also yielded no evidence of effect (SMD 0.39, 95% CI -0.17 to 0.94) [72]. There have also been systematic reviews with contradicting results: Butler and colleagues (2013) examined the effect of anodal tDCS on upper limb motor recovery in people with stroke and included randomised controlled trials, non-randomised trials and pre–post trials. Their analyses revealed a statistically significant beneficial effect of tDCS on upper limb function (SMD 0.49, 95% CI 0.18 to 0.817; seven studies with 168 participants) [73]. One reason for the discrepancy between their results and ours might be that the authors also included non-randomised studies, and that their meta-analyses suffered from multiplicity.

We found evidence of an effect of tDCS for improving ADL capacity, but not for improving arm function. Since there is only a weak association between paresis of one upper limb after stroke and ADL scores, one could argue that the improvement in ADL capacity may be not based on an improvement of the paretic arm itself, but rather on a generalized treatment effect, or on chance.

A recent systematic review with pairwise meta-regression explored several stimulation variables, like electrode size, electric current, current density, tDCS duration, number of sessions, electric charge, total electric charge, and total electric charge density [74]. The authors included ten comparisons of eight RCTs with 213 participants which measured arm function after stroke. They identified pad size, charge density, and current density as potentially relevant effect modifiers in studies measuring arm function by UE-FM, by entering each of the variables in an inverse variance-weighted linear meta-regression. We incorporated 19 comparisons of 16 studies with 302 participants measuring arm function by UE-FM, and could not find any statistically significant potential effect modifier. This might be explained by our different sample as well as our different approach to data extraction and meta-regression analysis.

To our knowledge, our review, including 26 genuine RCTs with a total of 754 participants, is the most comprehensive review so far of the effects and safety of tDCS regarding ADL capacity and arm function. However, our study has several limitations. These concern the level of individual studies and outcomes in the review as well as that of the review itself. At the level of individual studies, there is the concern about overestimating treatment effects and safety due to unclear or sometimes even high risk of bias, and the fact that the reporting of adverse events was often unsatisfactory. However, our sensitivity analysis regarding methodological quality was in accordance with the results of our main analysis. Another aspect is that there was methodological and clinical heterogeneity among the included studies regarding study type (the majority of included studies were phase II studies with rather small sample sizes, hence prone to the risk of baseline imbalance), age of the participants, time since stroke, dosage of stimulation, electrode location, base therapy (i.e., concurrent treatment) and level of initial severity. This may be due to the fact that the optimal stimulation paradigm still has to be established, since theoretical assumptions about the interaction between motor learning and tDCS-enhanced brain plasticity are still weak. This includes the optimal electrode placement. In popular electrode settings most of the current is redirected by the skin covering the skull, hence being unable to ‘trigger’ neurons effectively [14]. Although tDCS easily could be coupled with novel technologies like, for example robot-assisted training, its added value to rehabilitation outcomes has been limited so far [15]. The bimodal balance recovery model might represent a further step towards a patient-tailored approach to tDCS. But if an interaction effect is assumed between motor learning (base therapy) and brain plasticity (tDCS), tDCS should start earlier. This, however, was not supported by our data.

All clinical trials did employ a simplistic dose strategy of tDCS, assuming increased or decreased excitability of stimulated brain areas under the anodal and cathodal electrode, respectively (a detailed qualitative description of interventions can be found in Additional file 2). However, recent dose-response studies suggest that anodal or cathodal tDCS follows a complex, non-linear intensity-dependent effect on neuronal networks [10, 75]. The electric fields induced by tDCS applied in current doses in humans are found not sufficient in themselves to trigger spikes but rather to activate neurons at subthreshold level [10, 64]. Current animal studies rather suggest that cathodal and anodal tDCS may respectively, introduce dendritic hyper- or depolarization of neural membranes [10, 64]. The tDCS induced polarization of membranes of the apical dendrite will differ from that of soma and basal dendrite and dependent on the direction (i.e., inward (anodal) or outward (cathodal) current [10]. In other words, the localization of hyper-and depolarization in the cortex will differ in the same neuron dependent on its cellular composition and its position in relation with the cortex surface. As a consequence, there is now strong evidence that tDCS may induce long-term potentiation (LTP) and long-term depression (LTD) of stimulated neuronal pools [10, 64], which are fundamental for Hebbian and non-Hebbian forms of neuronal plasticity [76]. Furthermore, anodal tDCS induced LTP may enhance the secretion of brain derived nerve growth factors (BDNF) such as GAP43 [77, 78], change interneuronal activity and metabolism of glia cells [10]. The complexity of neuromodulation by tDCS suggests that a more sophisticated approach of tDCS is required to target neural networks effectively in a functional way [10].

Regarding the review level, there is the concern about violating the transitivity assumption, which means that included studies lack comparability. Violating the assumption of transitivity is more likely in larger treatment networks or in systematically different study conditions, like a wide variation in dates of study performance [24]. Neither of these was the case in our analyses. Although our formal analyses regarding inconsistency in the treatment networks were negative, this does not automatically mean that no inconsistency occurred [21]. Another point is that network meta-analyses require reasonably homogeneous studies, which is why we restricted our analysis to the post-intervention effects of tDCS. Since stroke is often a chronic disease, future network meta-analyses could also focus on the sustainability of effects of cathodal tDCS, acknowledging that the number of published trials that included long-term outcomes is rather small.

## Conclusions

Our network meta-analysis of randomised controlled trials suggests that cathodal tDCS is the most promising treatment option when tDCS is used to improve ADL capacity and arm function in people with stroke. There is evidence of an effect of cathodal tDCS in terms of improving ADL capacity. There is no evidence of an effect of either cathodal or any other tDCS stimulation type in terms of improving the function of the upper paretic limb after stroke, as measured by UE-FM. No difference regarding safety (in terms of dropouts and adverse events) was seen between different types of tDCS and their control interventions.

Next to improve the methodological quality of thee proof-of-concept trials, future trials in humans need to improve reporting the exact dose of tDCS including the electrode montage (electrode size and position) allowing to replicate findings [9]. In particular, the present meta-analysis shows that tDCS trials should improve the methodological quality of research. In particular there is room for improvement with respect to allocation concealment, report of drop outs and accompanying intention-to-treat analyses, as well as report of adverse events and long-term outcomes post intervention.Finally, the current network meta-analysis also suggests that the scientific rigor of different types of tDCS hamper and require a better understanding of its underlying working mechanism [64].

This finding suggests that not only methodological caveats but also technical limitations and insufficient fundamental knowledge about the dose-dependent working mechanism of tDCS may have influenced the current scientific rigor of this promising therapy.

## Additional files

Additional file 1: Search strategy for MEDLINE. (PDF 48 kb)

Additional file 2: Characteristics of included studies. (PDF 92 kb)

Additional file 3: Risk of bias of included studies. (PDF 101 kb)

Additional file 4: Presentation of outcomes of included studies. (PDF 90 kb)

Additional file 5: Inconsistency tables and net heat plots. (PDF 70 kb)

Additional file 6: Results of sensitivity analyses. (PDF 123 kb)

Additional file 7: Results of post hoc meta-regression. (PDF 86 kb)

## Abbreviations

95% CI: 95% confidence interval
ADL: Activities of daily living
BDNF: Brain derived nerve growth factors
LTD: Long-term depression
LTP: Long-term potentiation
M1: Primary motor cortex
MD: Mean difference
NIBS: Non-invasive brain stimulation
NMA: Network meta-analysis
RD: Risk difference
rTMS: Repetitive transcranial magnetic stimulation
SMD: Standardised mean difference
SUCRA: Surface under the cumulative ranking curve
tACS: Transcranial alternating current stimulation
tDCS: Transcranial direct current stimulation
TPU: Transcranial pulsed ultrasound
UE-FM: Upper extremity Fugl-Meyer assessment
