# Supplementary material for: Transcranial direct current stimulation (tDCS) for improving capacity in activities and arm function after stroke: a network meta-analysis of randomised controlled trials
Source: J Neuroeng Rehabil. 2017 Sep 13;14:95. doi: 10.1186/s12984-017-0301-7 (PMC5598049; doi:10.1186/s12984-017-0301-7)
Supplement: Supplementary file 1 — Search strategy for MEDLINE. (PDF 48 kb) [file 12984_2017_301_MOESM1_ESM.pdf]

Additional file 1: search strategy for searching MEDLINE via Ovid

1. cerebrovascular disorders/ or exp basal ganglia cerebrovascular disease/ or exp brain ischemia/ or exp carotid artery diseases/ or exp intracranial arterial diseases/ or exp "intracranial embolism and thrombosis"/ or exp intracranial hemorrhages/ or stroke/ or exp brain infarction/ or vertebral artery dissection/
2. (stroke or poststroke or post-stroke or cerebrovasc$ or brain vasc$ or cerebral vasc$ or cva$ or apoplex$ or SAH).tw.
3. ((brain$ or cerebr$ or cerebell$ or intracran$ or intracerebral) adj5 (isch?emi$ or infarct$ or thrombo$ or emboli$ or occlus$)).tw.
4. ((brain$ or cerebr$ or cerebell$ or intracerebral or intracranial or subarachnoid) adj5 (haemorrhage$ or hemorrhage$ or haematoma$ or hematoma$ or bleed$)).tw.
5. hemiplegia/ or exp paresis/
6. (hemipleg$ or hemipar$ or paresis or paretic or hemineglect or hemi-neglect or ((unilateral or spatial or hemi?spatial or visual) adj5 neglect)).tw.
7. or/1-6
8. Electric Stimulation Therapy/
9. Electric Stimulation/
10. Electrodes/
11. (transcranial adj5 direct current adj5 stimulation).tw.
12. (transcranial adj5 DC adj5 stimulation).tw.
13. (transcranial adj5 electric$ adj5 stimulation).tw.
14. (tDCS or A-tDCS or C-tDCS or S-tDCS or electrode$ or anode or anodes or anodal or cathode or cathodes or cathodal).tw.
15. or/8-14
16. Randomized Controlled Trials as Topic/
17. random allocation/
18. Controlled Clinical Trials as Topic/
19. control groups/
20. clinical trials as topic/ or clinical trials, phase i as topic/ or clinical trials, phase ii as topic/ or clinical trials, phase iii as topic/ or clinical trials, phase iv as topic/
21. double-blind method/
22. single-blind method/
23. Placebos/
24. placebo effect/
25. cross-over studies/
26. randomized controlled trial.pt.
27. controlled clinical trial.pt.
28. (clinical trial or clinical trial phase i or clinical trial phase ii or clinical trial phase iii or clinical trial phase iv).pt.
29. (random$ or RCT or RCTs).tw.
30. (controlled adj5 (trial$ or stud$)).tw.
31. (clinical$ adj5 trial$).tw.
32. ((control or treatment or experiment$ or intervention) adj5 (group$ or subject$ or patient$)).tw.
33. (quasi-random$ or quasi random$ or pseudo-random$ or pseudo random$).tw.
34. ((control or experiment$ or conservative) adj5 (treatment or therapy or procedure or manage$)).tw.
35. ((singl$ or doubl$ or tripl$ or trebl$) adj5 (blind$ or mask$)).tw.
36. (cross-over or cross over or crossover).tw.
37. (placebo$ or sham).tw.
38. trial.ti.
39. (assign$ or allocat$).tw.
40. controls.tw.
41. or/16-40
42. 7 and 15 and 41

43. exp animals/ not humans.sh.
44. 42 not 43
