# Supplementary material for: Transcranial direct current stimulation (tDCS) for improving capacity in activities and arm function after stroke: a network meta-analysis of randomised controlled trials
Source: J Neuroeng Rehabil. 2017 Sep 13;14:95. doi: 10.1186/s12984-017-0301-7 (PMC5598049; doi:10.1186/s12984-017-0301-7)
Supplement: Supplementary file 2 — Characteristics of included studies. (PDF 92 kb) [file 12984_2017_301_MOESM2_ESM.pdf]

Additional file 2: characteristics of studies examining the effects of tDCS on activities, arm function and acceptability

Study-ID	Design and sample size	EXP mean age (SD), years	CTL mean age (SD), years	EXP mean time post-stroke (SD)	CTL mean time post-stroke (SD)	Stimulation variables	Control Intervention	Base treatment	Outcomes
Allman et al., 2016 [44]	RCT; Anodal: 11 Sham: 13	67 (10)	60 (12)	57 (40) months	51 (33) months	20 min with 1 mA of anodal tDCS with the anode placed over the M1 of the affected hemisphere<br>Reference electrode position: over the contralateral supraorbital forehead<br>Treatment was delivered on 9 consecutive working days	Sham tDCS (1 mA for 10 s)	Graded Repetitive Arm Supplementary Program (GRASP) for 60 min on 9 consecutive working days	ARAT, WMFT, UE-FM, fMRI
Ang et al., 2012 [60]	RCT; Dual: 10 Sham: 9	52 (12)	56 (10)	3 (2) years	3 (1) years	20 min with 1 mA of dual tDCS with the anode placed over the M1 of the affected hemisphere and the cathode placed over the M1 of the unaffected hemisphere (size not stated)<br>Stimulation was delivered 5 times a week for 2 weeks	Sham tDCS (1 mA for 30 s)	60 min of therapy with the MIT-Manus arm robot 5 times a week for 2 weeks	UE-FM
Au-Yeung et al., 2014 [61]	Randomised cross-over trial; Anodal: 10 Cathodal: 10 Sham: 10	63 (6)		8 (3) years		Anodal: 20 min with 1 mA with 35 cm² anodal electrode over the M1 of the lesioned hemisphere<br>Cathodal: 20 min with 1 mA with 35 cm² cathodal electrode over the M1 of the non-lesioned hemisphere<br>Reference electrode position: over the contralateral supraorbital forehead<br>Stimulation was delivered once with at least 5 days of resting period	Sham tDCS (1 mA for 10 s)	None	PPT, Pinch grip strength
Boggio et al., 2007 [50]	Randomised cross-over trial; Anodal: 2 Cathodal: 1 Sham: 1	56 (11)	75 (NA)	33 (34) months	39 (NA) months	Anodal: 20 min with 1 mA with 35 cm² anodal electrode over the M1 of the lesioned hemisphere<br>Cathodal: 20 min with 1 mA with 35 cm² cathodal electrode over the M1 of the non-lesioned hemisphere<br>Reference electrode position: over the contralateral supraorbital forehead<br>Stimulation was delivered once a day for 4 days	Sham tDCS (1 mA for 30 s)	None	JTT
Bolognini et al., 2011 [27]	RCT; Anodal: 10 Sham: 11	43 (13)	51 (15)	44 (31) months	26 (18) months	20 min with 1 mA with 35 cm² anodal electrode over the M1 of the lesioned hemisphere<br>Stimulation was delivered 5 days a week for 2 consecutive weeks	Sham tDCS (2 mA for 30 s)	CIMT up to 4 hours/day for 5 days a week for 2 consecutive weeks	JTT, handgrip strength, MAL, UE-FM, questionnai

									re for adverse effects
Cha et al., 2014 [39]	RCT; Anodal: 10 Active Control: 10	60 (11)	58 (10)	14 (5) months	15 (4) months	20 min with 1 mA with 35 cm² anodal electrode over the M1 of the lesioned hemisphere<br>Reference electrode position: over the contralateral supraorbital forehead<br>Stimulation was delivered 5 days a week for 4 weeks	Basic training for improving function of upper and lower extremities for 30 min a day, 5 times a week for 4 weeks		UE-FM, grip strength
DiLazzaro et al., 2014a [28]	RCT; Dual: 7 Sham: 7	66 (16)	71 (14)	3 (1) days	3 (1) days	40 min with 2 mA with 35 cm² with the anode over the M1 of the lesioned hemisphere and cathode over the M1 of the non-lesioned hemisphere<br>Stimulation was delivered 5 on consecutive days	Sham tDCS (2 mA for 30 s)	None	ARAT, 9HPT, handgrip strength, MAL, NIHSS, mRS, adverse events
DiLazzaro et al., 2014b [29]	RCT; Dual: 10 Sham: 10	61 (16)	69 (12)	3 (2) days	3 (1) days	40 min with 2 mA with 35 cm² with the anode over the M1 of the lesioned hemisphere and cathode over the M1 of the non-lesioned hemisphere<br>Stimulation was delivered 5 on consecutive days	Sham tDCS (2 mA for 30 s)	CIMT for at least 90% of waking hours, including 1.5 hours a day arm training	ARAT, 9HPT, handgrip strength, MAL, NIHSS, mRS, adverse events, functional imaging data
Fregni et al., 2005 [62]	Randomised cross-over trial; Anodal: 6 Cathodal:6 Sham: 6	54 (17) years		27 (24) months		Anodal: 20 min with 1 mA with 35 cm² anodal electrode over the M1 of the lesioned hemisphere<br>Cathodal: 20 min with 1 mA with 35 cm² cathodal electrode over the M1 of the non-lesioned hemisphere<br>Stimulation was delivered once with at least 48 h resting period	Sham tDCS (1 mA for 30 s)	None	JTT
Fusco et al., 2013 [51]	Randomised cross-over trial; Anodal: 9 Cathodal: 9 Dual: 9 Sham: 9	44 (16)	65 (22)	31 (13) days	25 (5) days	Anodal: 15 min with 1.5 mA with 35 cm² anodal electrode over the M1 of the lesioned hemisphere<br>Cathodal: 15 min with 1.5 mA with 35 cm² cathodal electrode over the M1 of the non-lesioned hemisphere<br>Dual: 15 min with 1.5 mA with 35 cm² with the anode over the M1 of the lesioned hemisphere and cathode over the M1 of the	Sham tDCS (not described by the authors)	None	9HPT-index, maximum pinch and grasp force, patient satisfaction

						non-lesioned hemisphere<br>Reference electrode position: over the contralateral supraorbital forehead<br>Active stimulation and sham stimulation were delivered alternatingly on 2 consecutive days			
Fusco et al., 2014 [40]	RCT;<br>Cathodal: 5<br>Sham: 6	56 (15)	60 (12)	19 (8) days		10 min with 1.5 mA with 35 cm² cathodal electrode over the M1 of the non-lesioned hemisphere<br>Reference electrode position: above the right shoulder<br>Stimulation was delivered 5 times a week for 2 weeks	Sham tDCS (not described by the authors)	45 min of individualised motor rehabilitation focusing on recovery of upper limb twice a day	Canadian Neurological Scale, BI, 9HPT, grasp and pinch force, UE-FM, RMI
Hesse et al., 2011 [30]	RCT;<br>Anodal: 32<br>Cathodal: 32<br>Sham: 32	65 (10)	66 (10)	4 (2) weeks	4 (2) weeks	Anodal: 20 min with 2 mA with 35 cm² anodal electrode over the M1 of the lesioned hemisphere<br>Cathodal: 20 min with 2 mA with 35 cm² cathodal electrode over the M1 of the non-lesioned hemisphere<br>Reference electrode position: over the contralateral supraorbital forehead<br>Stimulation was delivered 5 days a week for 6 consecutive weeks	Sham tDCS (0 mA for 20 s)	20 min of robot-assisted arm training with the Bi-Manu-Track system 5 days a week for 6 consecutive weeks	UE-FM, BI, muscle strength, muscle tone, BBT
Khedr et al., 2013 [31]	RCT;<br>Anodal: 14<br>Cathodal: 13<br>Sham: 13	59 (9)	57 (8)	13 (5) days	13 (5) days	Anodal: 25 min with 2 mA with 35 cm² anodal electrode over the M1 of the lesioned hemisphere<br>Cathodal: 25 min with 2 mA with 35 cm² cathodal electrode over the M1 of the non-lesioned hemisphere<br>Reference electrode position: over the contralateral supraorbital forehead<br>Stimulation was delivered for 6 consecutive days	Sham tDCS (2 mA for 2 min)	Passive movement and range of motion exercise up to active resistive exercise within 1 hour after tDCS	NIHSS, OMCASS, BI, muscle strength, cortical excitability
Kim et al., 2009 [63]	Randomised cross-over trial;<br>Anodal: 10<br>Sham: 10	63 (13)		6 (3) weeks		20 min with 1 mA with 35 cm² anodal electrode over the M1 of the lesioned hemisphere<br>Reference electrode position: over the contralateral supraorbital forehead<br>Stimulation was delivered once, separated by at least 24 h of resting period	Sham tDCS (1 mA for 30 s)	None	BBT, VAS for fatigue
Kim et al., 2010 [32]	RCT;<br>Anodal: 7<br>Cathodal:6<br>Sham: 7	54 (15)	63 (9)	27 (21) days	23 (8) days	Anodal: 20 min with 2 mA with 35 cm² anodal electrode over the M1 of the lesioned hemisphere<br>Cathodal: 20 min with 2 mA with 35 cm² cathodal electrode over the M1 of the non-lesioned hemisphere<br>Reference electrode position: over the contralateral supraorbital forehead	Sham tDCS (2 mA for 1 min)	30 min of standardised occupational therapy to improve paretic hand function for 5 days a week for 2 consecutive weeks	UE-FM

						Stimulation was delivered 5 days a week for 2 consecutive weeks			
Lee et al., 2014 [33]	RCT;<br>Cathodal: 21<br>Active control: 22<br>Cathodal + active control: 21	62 (11)	61 (14)	18 (8) days	17 (6) days	20 min with 2 mA with 25 cm² cathodal electrode over the M1 of the non-lesioned hemisphere<br>Reference electrode position: over the contralateral supraorbital forehead<br>Stimulation was delivered 5 days a week for 3 weeks	Virtual reality solely for 30 minutes a day, 5 times a week for 3 weeks	Cathodal: occupational therapy for 30 min a day, 5 times a week for 3 weeks<br>Cathodal + active control and virtual reality only groups: virtual reality therapy for 30 min a day, 5 times a week for 3 weeks	MAS, MMT, UE-FM, K-MBI
Lindenberg et al., 2010 [41]	RCT;<br>Dual: 10<br>Sham: 10	62 (15)	56 (13)	31 (21)	40 (23)	30 min with 1.5 mA with 16.3 cm² anode over the M1 of the lesioned hemisphere and cathode over the M1 of the non-lesioned hemisphere<br>Stimulation was delivered on 5 consecutive days	Sham tDCS (1.5 mA for 30 s)	60 min of physical and occupational therapy on 5 consecutive days, including functional motor tasks	UE-FM, WMFT
Mahmoudi et al., 2011 [64]	Randomised cross-over trial;<br>Anodal A: 10<br>Anodal B: 10<br>Cathodal: 10<br>Dual: 10<br>Sham: 10	61 (14)		8 (5) months		Anodal A: 20 min with 1 mA with 35 cm² anodal electrode over the M1 of the lesioned hemisphere and the reference electrode over the contralateral orbit<br>Anodal B: 20 min with 1 mA with 35 cm² anodal electrode over the M1 of the lesioned hemisphere and the reference electrode on the contralateral deltoid muscle<br>Cathodal: 20 min with 1 mA with 35 cm² cathodal electrode over the M1 of the non-lesioned hemisphere and the reference electrode on the contralateral deltoid muscle<br>Dual: 20 min with 1 mA with 35 cm² with the anode over the M1 of the lesioned hemisphere and cathode over the M1 of the non-lesioned hemisphere<br>Each stimulation type was delivered once with at least 96 h resting period.	Sham tDCS (1 mA for 30 s)	None	JTT
Mortensen et al., 2016 [53]	RCT;<br>Anodal: 8<br>Sham: 7	66 (11)	61 (10)	32 (16) months	29 (15) months	20 min with 1 mA with 35 cm² anodal electrode over the M1 of the lesioned hemisphere and the reference electrode over the contralateral orbit<br>Treatment was delivered on 5 consecutive days	Sham tDCS (30 s with 1 mA max.)	30 min of home-based occupational therapy	JTT, grip strength, SIS
Nair et al., 2011 [47]	RCT;<br>Cathodal: 7<br>Sham: 7	61 (12)	56 (15)	33 (20) months	28 (28) months	30 min with 1 mA with cathodal electrode over the M1 of the non-lesioned hemisphere and the reference electrode on the contralateral deltoid muscle<br>Stimulation was delivered on 5 consecutive days	Sham tDCS (not described by the authors)	60 min of occupational therapy for 5 consecutive days	3J-ROM, change in UE-FM
Qu et al., 2009 [34]	RCT;<br>Cathodal: 25	45 (11)	45 (14)	6 (range 3 to 36) months	4 (range 3 to 12) months	20 min with 0.5 mA with cathodal electrode over the M1 of the non-lesioned hemisphere<br>Reference electrode position: unclear	Physical therapy according to the Bobath, Brunnstrom and Rood approaches for 40 min twice a day for 5 consecutive days for 4 weeks		BI, MAS, UE-FM

	Sham: 25					Stimulation was delivered 5 days a week for 4 weeks			
Rocha et al., 2016 [45]	RCT;<br>Anodal: 7<br>Cathodal: 7<br>Sham: 7	58 (range 41-71)	59 (range 46-70)	31 months (range 9-67)	27 months (6-46)	Anodal: 13 min with 1 mA with 35 cm² anodal electrode over the M1 of the lesioned hemisphere<br>Cathodal: 9 min with 1 mA with 35 cm² cathodal electrode over the M1 of the non-lesioned hemisphere<br>Reference electrode position: over the contralateral supraorbital forehead<br>Stimulation was delivered 5 days a week for 2 consecutive weeks	Sham tDCS (1 mA for 30 s)	Modified CIMT for 6 consecutive hours each day for 4 weeks plus 1 h gross and fine motor activities training per day	UE-FM, MAL, handgrip strength
Rossi et al., 2013 [42]	RCT;<br>Anodal: 25<br>Sham: 25	66 (14)	70 (14)	2 days	2 days	20 min with 2 mA with anodal electrode over the M1 of the lesioned hemisphere<br>Reference electrode position: over the contralateral supraorbital forehead<br>Stimulation was delivered on 5 consecutive days	Sham tDCS (2 mA for 30 s)	Not described by the authors	NIHSS, UE-FM
Sattler et al., 2015 [46]	RCT;<br>Anodal: 10<br>Sham: 10	68 (10)	63 (12)	5 (3) days	6 (4) days	13 min with 1.2 mA with anodal electrode over the M1 of the lesioned hemisphere<br>Reference electrode position: over the contralateral supraorbital forehead<br>Stimulation was delivered on 5 consecutive days	1.2 mA for 60 s	rPNS for 13 min on 5 consecutive days + occupational therapy 3 to 5 times a week for 30 min	JTT, handgrip strength, 9HPT, hand tapping test, UE-FM
Sik et al, 2015 [54]	RCT;<br>Anodal: 10<br>Dual: 10<br>Sham: 11	Median (25th-75th percentile):<br>Anodal: 62 (55-70)<br>Dual: 59 (54-66)	Median (25th-75th percentile): 60 (55-67)	Median (25th-75th percentile):<br>Anodal: 21 (8-44) months<br>Dual: 25 (9-38) months	Median (25th-75th percentile): 18 (12-33) months	Anodal: 20 min with 2 mA with 16 cm² anodal electrode over the M1 of the lesioned hemisphere<br>Dual: anodal + 20 min with 2 mA with 16 cm² cathodal electrode over the M1 of the non-lesioned hemisphere<br>Reference electrode position: over the contralateral supraorbital forehead<br>Treatment was delivered in 15 sessions during 3 weeks	2 mA for 1 min	15 sessions of physiotherapy and occupational therapy for 120 min during 3 weeks	JTT, WMFT, Kocaeli Functional Evaluation Test
Straudi et al., 2016 [37]	RCT;<br>Dual: 12<br>Sham: 11	53 (16)	64 (10)	41 (35) weeks	78 (62) weeks	30 min with 1 mA with 35 cm² anodal electrode over the M1 of the lesioned hemisphere and cathodal electrode over the M1 of the non-lesioned hemisphere	Sham tDCS (1 mA for 30 s)	30 min of robotic arm training with the REO device	UE-FM, BBT, MAL, adverse-effects questionnaire
Tedesco-Triccas et al., 2015 [35]	RCT;<br>Anodal: 12<br>Sham: 11	64 (10)	63 (14)	25 (31) months	13 (16) months	20 min with 1 mA with anodal electrode over the M1 of the lesioned hemisphere<br>Reference electrode position: over the contralateral supraorbital forehead<br>Stimulation was delivered on 18 sessions	Sham tDCS (1 mA for 20 s)	Robotic arm training with the ArmeoSpring device (60 min per session) for 18 sessions during 8 weeks (2 to 3 sessions a week)	UE-FM, ARAT, MAL, SIS 3.0

						during 8 weeks (between 2 and 3 sessions per week)			
Viana et al., 2014 [43]	RCT; Anodal: 10 Sham: 10	56 (10)	55 (12)	32 (18) months	35 (20) months	13 min with 1 mA with anodal electrode over the M1 of the lesioned hemisphere<br>Reference electrode position: over the contralateral supraorbital forehead<br>Stimulation was delivered 3 times a week for 5 weeks	Sham tDCS (2 mA for 30 s)	Virtual reality training using Nintendo Wii aiming at arm movements, for 60 min	UE-FM, WMFT, MAS, hand-held dynamometry
Wang et al., 2014 [52]	RCT; Anodal tDCS + Placebo methylphenidate: 3 Sham tDCS + methylphenidate: 3 Anodal + methylphenidate: 3	54 (14)	52 (9)	Not explicitly stated, but all participants were enrolled between 1 and 4 weeks post stroke		Dual: 20 min with 1 mA with 35 cm² with the anode over the M1 of the lesioned hemisphere and cathode over the M1 of the non-lesioned hemisphere<br>Reference electrode position: over contralateral M1<br>Stimulation was delivered once	Sham tDCS (1 mA for 10 s) and placebo methylphenidate	Placebo methylphenidate 1 hour prior to stimulation, active 20 mg methylphenidate 1 hour prior to stimulation	PPT, cortical excitability
Wu et al., 2013 [36]	RCT; Cathodal: 45 Sham: 45	46 (11)	49 (13)	5 (3) months	5 (3) months	20 min with 1.2 mA with 24.75 cm² cathodal electrode over the M1 of the non-lesioned hemisphere<br>Reference electrode position: over the shoulder on the unaffected side<br>Stimulation was delivered 5 days a week for 4 weeks	Sham tDCS (1.2 mA for 30 s)	conventional physical therapy program for 30 min twice daily 5 days a week for 4 weeks	MAS, UE-FM, MBI

ARAT: Action Research Arm Test, BBT: Box and Block Test, BI: Barthel Index, CIMT: constraint-induced movement therapy, CTL: Control group, EXP: Experimental group, JTT: Jebsen Taylor Hand Function Test, M1: primary motor cortex, mA: milliampere, MAL: Motor Activity Log Rating Scale, MAS: Modified Ashworth Scale, MBI: Modified Barthel Index, MI: Motricity Index, MIT: Massachusetts Institute of Technology, MRC: Medical Research Council, NIHSS: National Institute of Health Stroke Scale, OMCASS: Orgogozo MCA scale, PPT: Purdue Pegboard Test, RCT: randomised controlled trial, ROM: range of motion, RMI: Rivermead Mobility Index, SD: standard deviation, SIS: Stroke Impact Scale, tDCS: transcranial direct current stimulation, TUG: Timed Up and Go Test, UE-FM: Upper Extremity Fugl-Meyer Score, WMFT: Wolf Motor Function Test
