# Supplementary material for: Transcranial direct current stimulation (tDCS) for improving capacity in activities and arm function after stroke: a network meta-analysis of randomised controlled trials
Source: J Neuroeng Rehabil. 2017 Sep 13;14:95. doi: 10.1186/s12984-017-0301-7 (PMC5598049; doi:10.1186/s12984-017-0301-7)
Supplement: Supplementary file 3 — Risk of bias of included studies. (PDF 101 kb) [file 12984_2017_301_MOESM3_ESM.pdf]

Additional file 3: Risk of bias of included studies

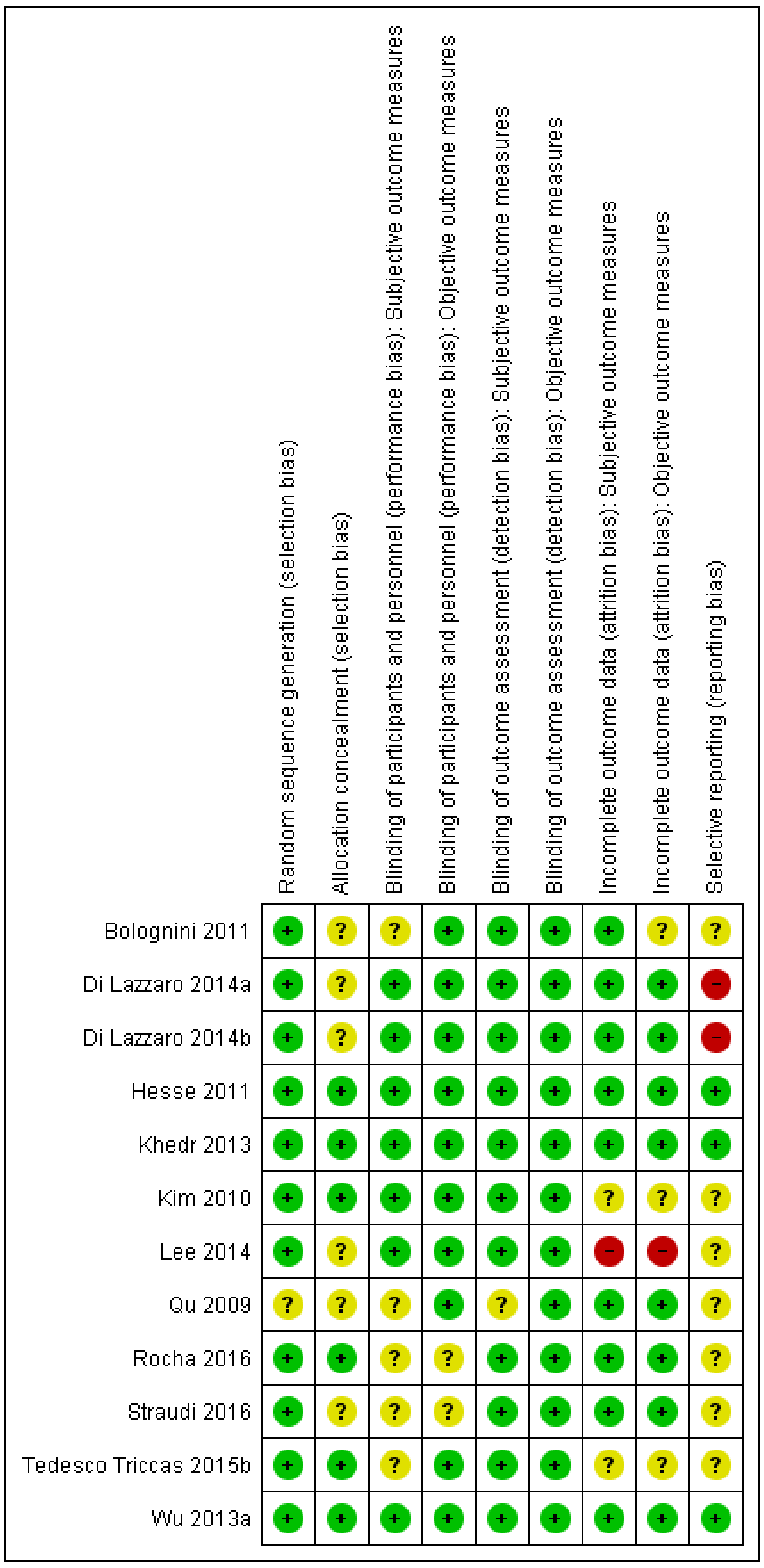

Risk of bias summary of tDCS for improving activities

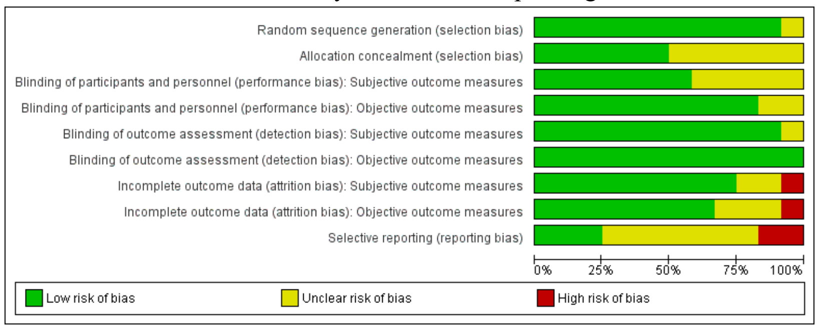

Risk of bias graph of tDCS for improving activities

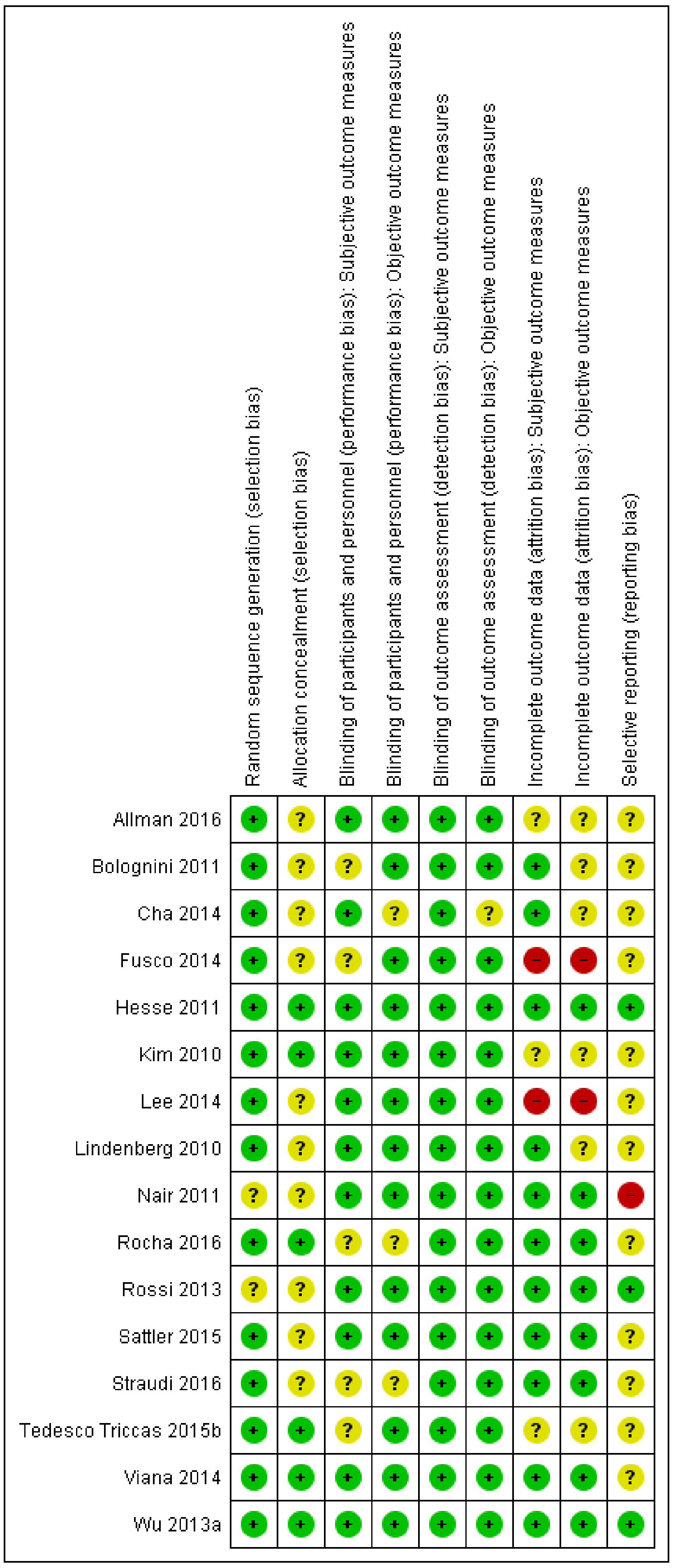

Risk of bias summary of tDCS for improving arm function

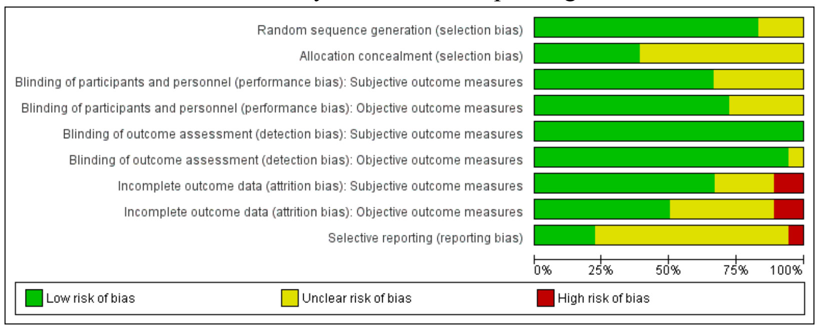

Risk of bias graph of tDCS for improving arm function

	Random sequence generation (selection bias)	Allocation concealment (selection bias)	Blinding of participants and personnel (performance bias): Subjective outcome measures	Blinding of participants and personnel (performance bias): Objective outcome measures	Blinding of outcome assessment (detection bias): Subjective outcome measures	Blinding of outcome assessment (detection bias): Objective outcome measures	Incomplete outcome data (attrition bias): Subjective outcome measures	Incomplete outcome data (attrition bias): Objective outcome measures	Selective reporting (reporting bias)
Allman 2016	+	?	+	+	+	+	?	?	?
Ang 2012	+	?	+	+	+	+	+	+	?
Boggio 2007a	?	?	+	+	+	+	+	+	?
Bolognini 2011	+	?	?	+	+	+	+	?	?
Cha 2014	+	?	+	?	+	?	+	?	?
Di Lazzaro 2014a	+	?	+	+	+	+	+	+	–
Di Lazzaro 2014b	+	?	+	+	+	+	+	+	–
Fusco 2013a	+	+	?	+	–	?	+	+	?
Fusco 2014	+	?	?	+	+	+	–	–	?
Hesse 2011	+	+	+	+	+	+	+	+	+
Khedr 2013	+	+	+	+	+	+	+	+	+
Kim 2010	+	+	+	+	+	+	?	?	?
Lee 2014	+	?	+	+	+	+	–	–	?
Lindenberg 2010	+	?	+	+	+	+	+	?	?
Mortensen 2016	+	+	?	?	+	+	+	+	+
Nair 2011	?	?	+	+	+	+	+	+	–
Qu 2009	?	?	?	+	?	+	+	+	?
Rocha 2016	+	+	?	?	+	+	+	+	?
Rossi 2013	?	?	+	+	+	+	+	+	+
Sattler 2015	+	?	+	+	+	+	+	+	?
Sik 2015	?	?	?	?	?	?	+	+	?
Straudi 2016	+	?	?	?	+	+	+	+	?
Tedesco Triccas 2015b	+	+	?	+	+	+	?	?	?
Viana 2014	+	+	+	+	+	+	+	+	?
Wang 2014	?	?	+	+	+	+	+	?	?
Wu 2013a	+	+	+	+	+	+	+	+	+

Risk of bias summary of the acceptability of tDCS

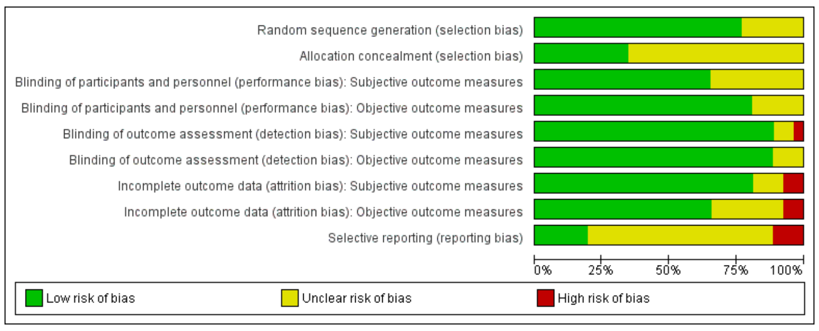

Risk of bias graph of the acceptability of tDCS
