# Supplementary material for: Transcranial direct current stimulation (tDCS) for improving capacity in activities and arm function after stroke: a network meta-analysis of randomised controlled trials
Source: J Neuroeng Rehabil. 2017 Sep 13;14:95. doi: 10.1186/s12984-017-0301-7 (PMC5598049; doi:10.1186/s12984-017-0301-7)
Supplement: Supplementary file 4 — Presentation of outcomes of included studies. (PDF 90 kb) [file 12984_2017_301_MOESM4_ESM.pdf]

Additional file 4: presentation of outcome data, by included study

tDCS for improving activities after stroke

Study	Assessment used	Anodal tDCS		Cathodal tDCS		Dual tDCS		Sham tDCS		Active control	
		Mean (SD)	Participants, *n*	Mean (SD)	Participants, *n*	Mean (SD)	Participants, *n*	Mean (SD)	Participants, *n*	Mean (SD)	Participants, *n*
Bolognini et al., 2011	MAL-QOM	2.0 (3.97)	10					2.5 (1.98)	11		
DiLazzaro et al., 2014a	mRS					3.0 (1)	7	3.0 (1.3)	7		
DiLazzaro et al., 2014b	mRS					2.4 (1.2)	10	3.1 (1.4)	10		
Hesse et al., 2011	BI	53.6 (14.5)	32	59.2 (12.4)	32			56.3 (15.5)	32		
Khedr et al., 2013	BI	52 (30)	14	52 (18)	13			41 (18)	13		
Kim et al., 2010	mBI	78 (14)	7	93 (18)	6			73 (19)	7		
Lee et al., 2014	mBI			69.9 (16.6)	42 (21+21)*					64.3 (24.5)	20
Qu et al., 2009	BI			74 (16)	25			74 (20)	25		
Rocha et al. 2016	MAL-QOM	3.19 (1.0)	7	3.19 (1.2)	7			2.65 (0.6)	7		
Straudi et al., 2016	MAL-QOM	1.05(1.43)	12					0.85 (1.50)	11		
Tedesco-Triccas et al., 2015	MAL-QOM	1.2 (2.1)	12					1.6 (1.8)	11		
Wu et al., 2013	MBI			76.2 (19.6)	45			65.4 (20.4)	45		

*Groups with similar interventions have been combined in a single group. BI: Barthel Index, MAL-QOM: Motor Activity Log Rating Scale, Quality of Movement subscale, MAL-AOU: Motor Activity Log Rating Scale, amount of use subscale, MAS: Modified Ashworth Scale, mBI: Modified Barthel Index, MI: Motricity Index, NIHSS: National Institute of Health Stroke Scale, OMCASS: Orgogozo MCA scale, RMI: Rivermead Mobility Index, SD: standard deviation, SIS: Stroke Impact Scale, tDCS: transcranial direct current stimulation

tDCS for improving arm function after stroke

Study	Assessment used	Anodal tDCS		Cathodal tDCS		Dual tDCS		Sham tDCS		Active control	
		Mean (SD)	Participants, *n*	Mean (SD)	Participants, *n*	Mean (SD)	Participants, *n*	Mean (SD)	Participants, *n*	Mean (SD)	Participants, *n*
Allman et al., 2016	UE-FM	50.4 (11.2)	11					45.5 (14.6)	13		
Bolognini et al., 2011	UE-FM	32 (37)	10					30 (33)	11		
Cha et al., 2014	UE-FM	48.7 (12.5)	10							29.7 (9.2)	10
Fusco et al., 2014	UE-FM change score			4 (5)	5			4 (7)	6		
Hesse et al., 2011	UE-FM	19.1 (14.4)	32	18.9 (10.5)	32			19.2 (15.0)	32		
Kim et al., 2010	UE-FM	56.7 (6.6)	7	61.0 (3.5)	6			41 (16.5)	7		
Lee et al., 2014	UE-FM			45.4 (22.3)	42 (21+21)*					41.6 (21.3)	20
Lindenberg et al., 2010	UE-FM					43.8 (12.3)	10	41.0 (11.8)	10		
Nair et al., 2011	UE-FM			33.7 (12.9)	7			32.3 (9.8)	7		
Rocha et al., 2016	UE-FM	11.2 (1.0)	7	7.3 (1.6)	7			3.9 (2.3)	7		
Rossi et al., 2013	UE-FM	7.5 (10.1)	25					9.6 (10.3)	25		
Sattler et al., 2015	UE-FM change score	6.6 (4.2)	10					9 (6.2)	10		
Straudi et al., 2016	UE-FM	28.5 (19.0)	12					26.6 (16.12)	11		
Tedesco-Triccas et al., 2015	UE-FM	33.6 (16.3)	12					44.8 (16.3)	11		
Viana et al., 2014	UE-FM	50.6 (13.4)	10					46.9 (12.4)	10		
Wu et al., 2013	UE-FM	22.3 (7.9)	45					14.6 (9.1)	45		

*Groups with similar interventions have been combined in a single group. ARAT: Action Research Arm Test, BBT: Box and Block Test, CIMT: constraint-induced movement therapy, UE-FM: Upper Extremity Fugl-Meyer Score

Safety of tDCS for improving activities or arm function after stroke, measured by number of dropouts and adverse events

Study	Anodal tDCS		Cathodal tDCS		Dual tDCS		Sham tDCS		Active control	
	Events, *n*	Participants, *n*	Events, *n*	Participants, *n*	Events, *n*	Participants, *n*	Events, *n*	Participants, *n*	Events, *n*	Participants, *n*
Allman et al., 2016	2	13					0	13		
Ang et al., 2012	0	10					0	9		
Bolognini et al., 2011	3	9					2	10		
Cha et al., 2014	0	10							0	10
DiLazzaro et al., 2014a					0	7	0	7		
DiLazzaro et al., 2014b					0	10	0	10		
Fusco et al., 2013	0	2	0	1	0	2	0	4		
Fusco et al., 2014			2	7			1	7		
Hesse et al., 2011	1	32	0	32	0	32				
Khedr et al., 2013	0	14	0	13			0	13		
Kim et al., 2010	1	7	1	6			0	7		
Lee et al., 2014			3	42 (21+21)*					1	21
Lindenberg et al., 2010					0	10	0	10		
Mortensen et al., 2016	0	8					1	7		
Nair et al., 2011			0	7			0	7		
Qu et al., 2009			0	25			0	25		
Rocha et al., 2016		7	0	7			0	7		
Rossi et al., 2013	0	25					0	25		
Sattler et al., 2015	0	10					0	10		
Sik et al, 2015	2	12			2	12	1	12		
Straudi et al., 2016	3	12					2	11		
Tedesco-Triccas et al., 2015	6	12					0	11		
Viana et al., 2014	0	10					0	10		
Wang et al., 2014	0	3					0	3		
Wu et al., 2013	0	45					0	45		

*Groups with similar interventions have been combined in a single group.
