# Supplementary material for: Transcranial direct current stimulation (tDCS) for improving capacity in activities and arm function after stroke: a network meta-analysis of randomised controlled trials
Source: J Neuroeng Rehabil. 2017 Sep 13;14:95. doi: 10.1186/s12984-017-0301-7 (PMC5598049; doi:10.1186/s12984-017-0301-7)
Supplement: Supplementary file 5 — Inconsistency tables and net heat plots. (PDF 70 kb) [file 12984_2017_301_MOESM5_ESM.pdf]

Additional file 5: Inconsistency tables and net heat plots

The columns and rows of the net heat plot correspond to the comparison of treatments within designs. Pairwise comparisons of three-arm studies are designated by "_". The area of the grey squares is proportional to the contribution made by the treatment comparison in the column to the treatment comparison in the row. If the largest grey square of a comparison is in the diagonal, this means that the direct comparison is the greatest source of information. The larger the size of the grey squares off the diagonal, the bigger the contribution of indirect evidence to the effect estimates. Cold-coloured comparisons indicate an increase in inconsistency and warm-coloured comparisons indicate a decrease in inconsistency by these comparisons.

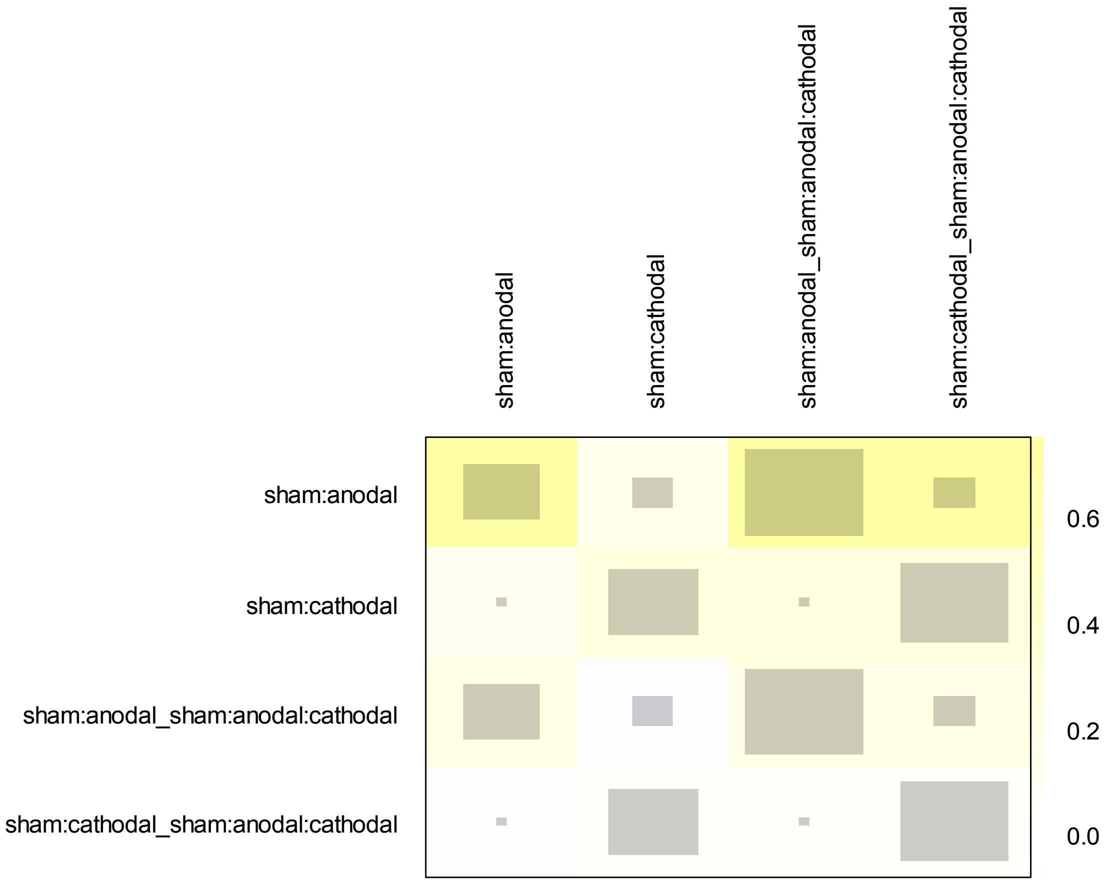

Net heat plot of tDCS for improving activities (random-effects model)

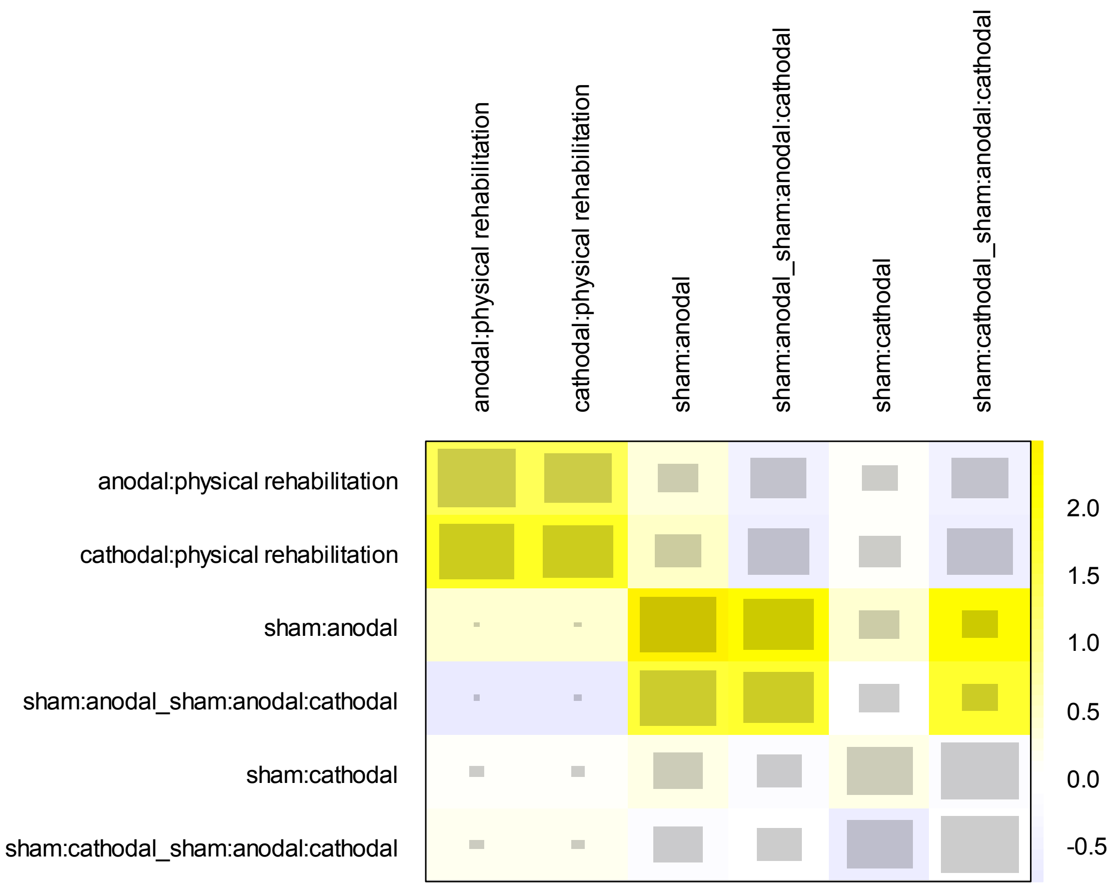

Net heat plot of tDCS for improving arm function (random-effects model)

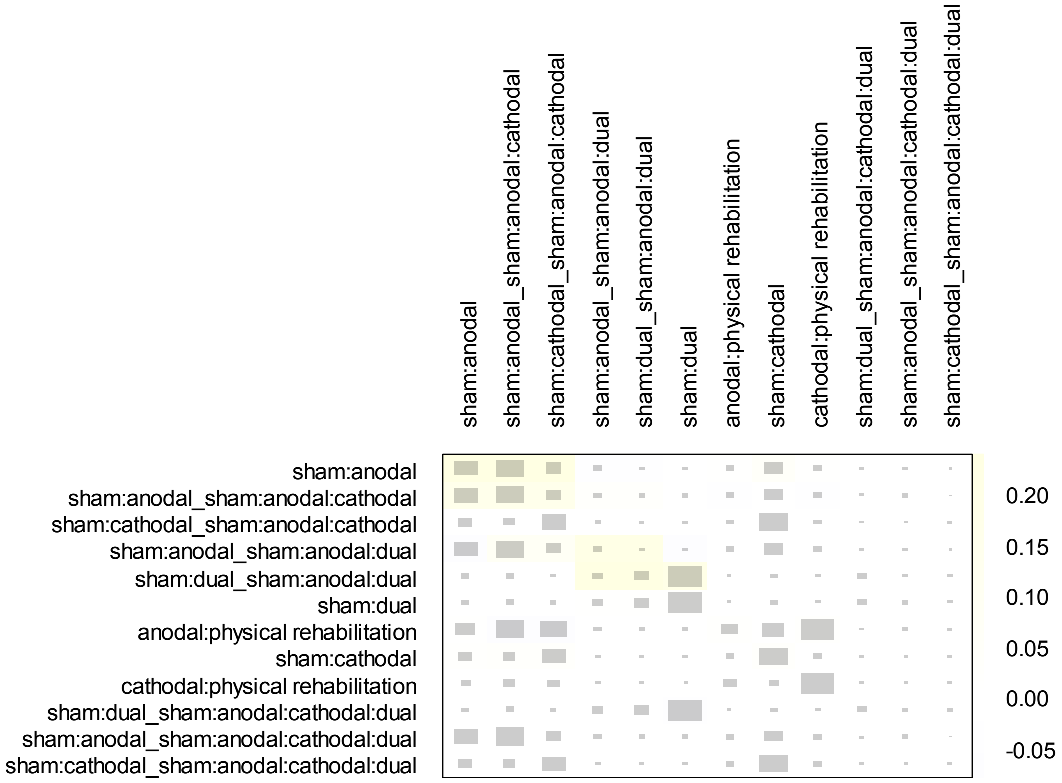

Net heat plot of the acceptability of tDCS for improving activities or arm function (random-effects model)
