# Supplementary material for: Transcranial direct current stimulation (tDCS) for improving capacity in activities and arm function after stroke: a network meta-analysis of randomised controlled trials
Source: J Neuroeng Rehabil. 2017 Sep 13;14:95. doi: 10.1186/s12984-017-0301-7 (PMC5598049; doi:10.1186/s12984-017-0301-7)
Supplement: Supplementary file 6 — Results of sensitivity analyses. (PDF 123 kb) [file 12984_2017_301_MOESM6_ESM.pdf]

Additional file 6: Results of sensitivity analyses

Results of sensitivity analysis regarding methodological quality for our primary outcome (activities)

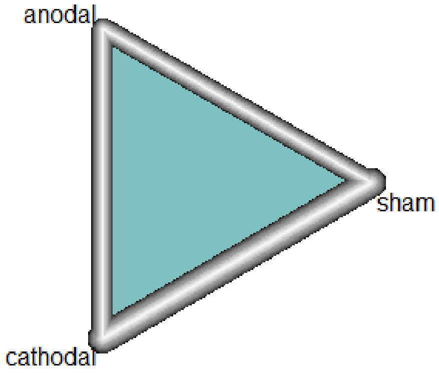

Network graph of tDCS for improving activities after stroke. The thicker the edge, the lower the standard error of this comparison. Coloured polygons indicate multi-arm studies.

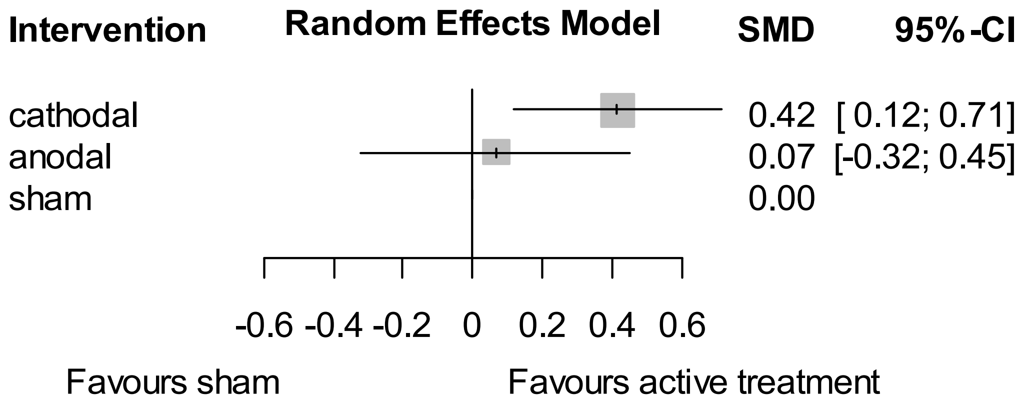

Forest plot of tDCS for improving ADL capacity after stroke (four studies with 247 participants). Treatments are listed in order of relative ranking. SMD = standardised mean difference, CI = confidence interval. Sham is the reference category.

Table AF5-1: treatment rankings by P-score of tDCS for improving ADL capacity (only studies with proper allocation concealment, blinding of outcome assessor, and intention-to-treat analysis)

**Treatment**	**P-Score**
Cathodal	0.98
Anodal	0.41
Sham	0.12

Treatments are listed in order of relative ranking. The P-Score describes the mean level of certainty about a particular treatment being better than another treatment.

Results of sensitivity analysis regarding the choice of fixed- or random-effects model

No systematic differences between a fixed- or random-effects model could be detected in our analyses by visually inspecting Bland-Altman plots.

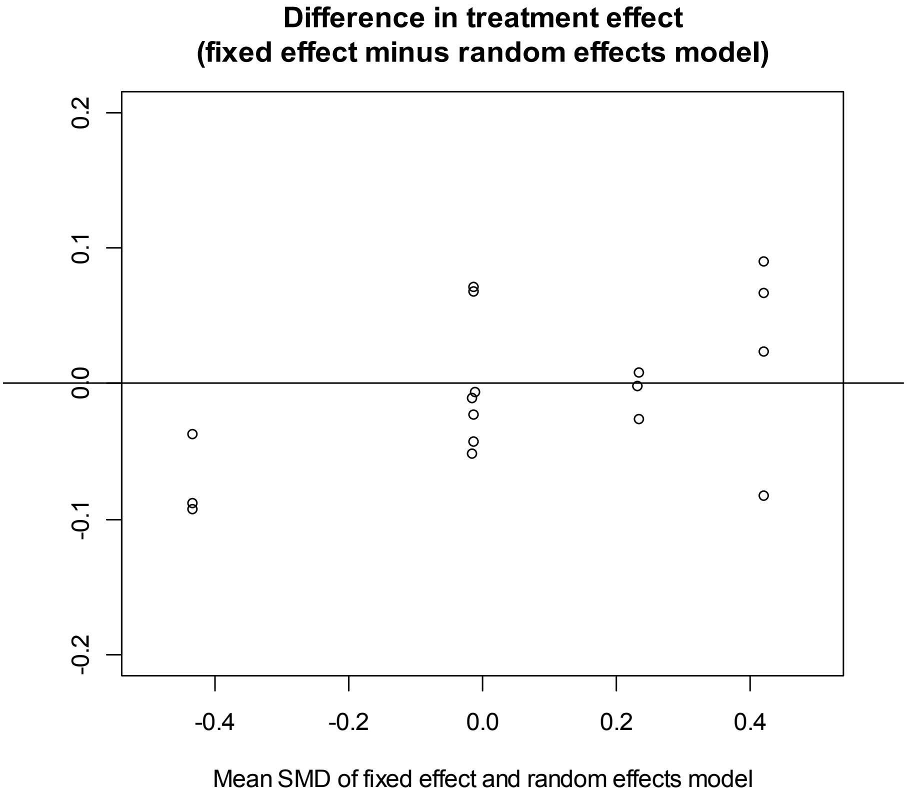

Bland-Altman plot of differences between fixed- and random-effects model in the treatment effects of tDCS for improving ADL capacity after stroke. Each circle represents a pairwise comparison.

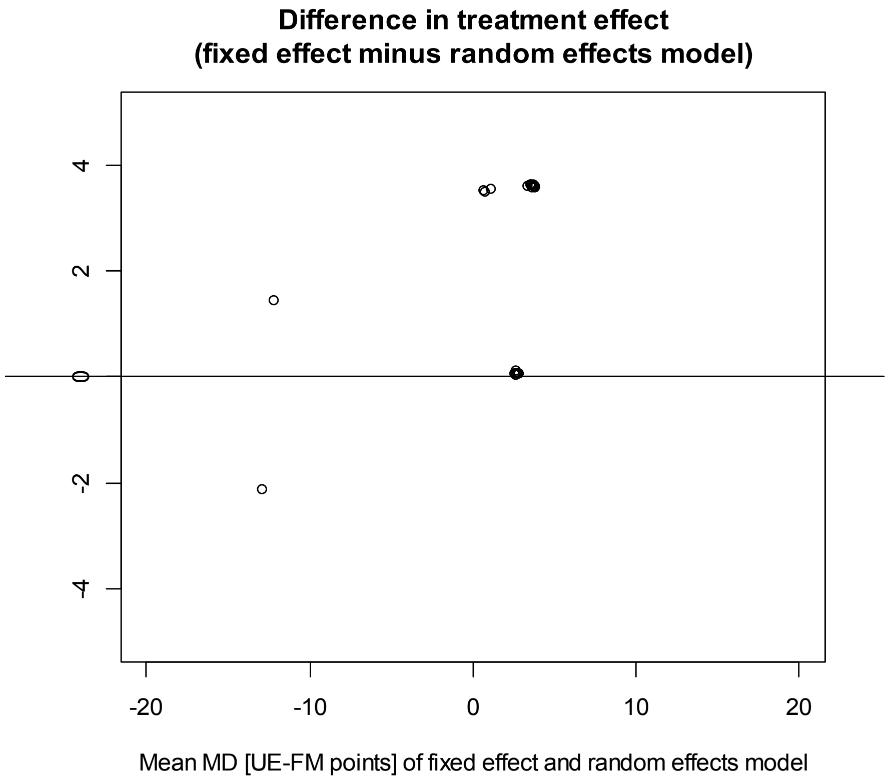

Bland-Altman plot of differences between fixed- and random-effects model in the treatment effects of tDCS for improving arm function after stroke (measured by UE-FM). UE-FM: Upper Extremity Fugl-Meyer Assessment. Each circle represents a pairwise comparison.
