# Supplementary material for: Transcranial direct current stimulation (tDCS) for improving capacity in activities and arm function after stroke: a network meta-analysis of randomised controlled trials
Source: J Neuroeng Rehabil. 2017 Sep 13;14:95. doi: 10.1186/s12984-017-0301-7 (PMC5598049; doi:10.1186/s12984-017-0301-7)
Supplement: Supplementary file 7 — Results of post hoc meta-regression. (PDF 86 kb) [file 12984_2017_301_MOESM7_ESM.pdf]

Additional file 7: Results of post-hoc meta-regression regarding publication year, time post-stroke, applied current [mA], duration of stimulation session [min], number of stimulation sessions, electrode size [cm²], current density [mA/cm²], electric charge per session [C], and electric charge density per session [C/cm²]

Meta-regression on primary outcome, ADL capacity

Extracted and calculated data from included sham-controlled trials examining the effects of tDCS on ADL capacity.

Study ID, Publication Year	Treatment	Time since stroke [months]	Applied current [mA]	Duration of stimulation session [min]	Number of stimulation sessions	Electrode size [cm²]	Current density [mA/cm²]	Charge per session [C]	Charge density per session [C/cm²]
Bolognini 2011	anodal vs. sham	35.21	2	40	10	35	0.06	4.8	0.14
DiLazzaro 2014a	dual vs. sham	0.09	2	40	5	35	0.06	4.8	0.14
DiLazzaro 2014b	dual vs. sham	0.11	2	40	5	35	0.06	4.8	0.14
Hesse 2011	anodal vs. sham	0.83	2	20	30	35	0.06	2.4	0.07
Hesse 2011	cathodal vs. sham	0.87	2	20	30	35	0.06	2.4	0.07
Khedr 2013	anodal vs. sham	0.43	2	25	6	35	0.06	3.0	0.09
Khedr 2013	cathodal vs. sham	0.41	2	25	6	35	0.06	3.0	0.09
Kim 2010	anodal vs. sham	0.92	2	20	10	25	0.08	2.4	0.10
Kim 2010	cathodal vs. sham	0.70	2	20	10	25	0.08	2.4	0.10
Rocha 2016	anodal vs. sham	27.00	1	13	20	35	0.03	0.8	0.02
Rocha 2016	cathodal vs. sham	30.35	1	9	20	35	0.03	0.5	0.02
Straudi 2016	dual vs. sham	13.49	1	30	10	35	0.03	1.8	0.05
Tedesco-Triccas 2015	anodal vs. sham	19.61	1	20	18	35	0.03	1.2	0.03
Wu 2013	cathodal vs. sham	4.90	1.2	20	20	24.75	0.05	1.4	0.06

C: Coulomb; cm²: square centimetre; mA: milliampere, min: minutes, SE: standard error

Variables derived from meta-regression model of tDCS for improving ADL capacity.

Variable	β	SE	P Value
Intercept	-1274.10	1257.51	0.31
Publication year	0.63	0.62	0.31
Time since stroke [months]	0.04	0.05	0.44
Applied current [mA]	2.05	5.17	0.69
Duration of stimulation session [min]	0.08	0.15	0.58
Number of stimulation sessions	0.02	0.04	0.69
Electrode size [cm²]	-0.11	0.26	0.67
Current density [mA/cm²]	90.41	171.02	0.60
Charge per session [C]	-0.50	2.84	0.86
Charge density per session [C/cm²*session]	-22.72	120.70	0.85

C: Coulomb; cm²: square centimetre; mA: milliampere, min: minutes, SE: standard error

Meta-regression on secondary outcome, arm function.

Extracted and calculated data from included sham-controlled trials examining the effects of tDCS on arm function as measured by Upper Extremity Fugl-Meyer assessment.

Study-ID, Publication Year	Treatment	Time since stroke [months]	Applied current [mA]	Duration of stimulation session [min]	Number of stimulation sessions	Electrode size [cm²]	Current density [mA/cm²]	Charge per session [C]	Charge density per session [C/cm²]
Allman 2016	anodal vs. sham	54.13	1	20	9	35	0.03	1.2	0.03
Bolognini 2011	anodal vs. sham	35.21	2	40	10	35	0.06	4.8	0.14
Fusco 2014	cathodal vs. sham	0.63	1.5	10	10	35	0.04	0.9	0.03
Hesse 2011	anodal vs. sham	0.83	2	20	30	35	0.06	2.4	0.07
Hesse 2011	cathodal vs. sham	0.87	2	20	30	35	0.06	2.4	0.07
Kim 2010	anodal vs. sham	0.92	2	20	10	25	0.08	2.4	0.10
Kim 2010	cathodal vs. sham	0.70	2	20	10	25	0.08	2.4	0.10
Lindenberg 2010	dual vs. sham	35.40	1.5	30	5	16.3	0.09	2.7	0.17
Nair 2011	cathodal vs. sham	30.50	1	30	5	NA	NA	1.8	NA
Rocha 2016	anodal vs. sham	27.00	1	13	20	35	0.03	0.8	0.02
Rocha 2016	cathodal vs. sham	30.35	1	9	20	35	0.03	0.5	0.02
Rossi 2013	anodal vs. sham	0.32	2	20	5	35	0.06	2.4	0.07
Sattler 2015	anodal vs. sham	0.18	1.2	13	5	35	0.03	0.9	0.03
Straudi 2016	dual vs. sham	13.49	1	30	10	35	0.03	1.8	0.05
Tedesco-Triccas 2015	anodal vs. sham	19.61	1	20	18	35	0.03	1.2	0.03
Viana2014	anodal vs. sham	33.45	2	13	15	35	0.06	1.6	0.04
Wu 2013	cathodal vs. sham	4.90	1.2	20	20	24.75	0.05	1.4	0.06

C: Coulomb; cm²: square centimetre; mA: milliampere, min: minutes, NA: not available, SE: standard error

Variables derived from meta-regression model of tDCS for improving arm function.

Variable	β	SE	P Value
Intercept	-3537.66	7120.91	0.62
Publication year	1.79	3.54	0.61
Time since stroke [months]	0.16	0.14	0.27
Applied current [mA]	29.92	33.66	0.37
Duration of stimulation session [min]	-0.29	0.89	0.74
Number of stimulation sessions	0.17	0.29	0.54
Electrode size [cm²]	-2.57	1.32	0.05
Current density [mA/cm²]	-758.41	865.13	0.38
Charge per session [C]	4.83	14.45	0.74
Charge density per session [C/cm²*session]	-38.66	292.23	0.89

C: Coulomb; cm²: square centimetre; mA: milliampere, min: minutes, SE: standard error
